# Typifying conservation practitioners’ views on the role of education

**DOI:** 10.1111/cobi.13893

**Published:** 2022-02-21

**Authors:** Aina Brias‐Guinart, Kaisa Korhonen‐Kurki, Mar Cabeza

**Affiliations:** ^1^ Global Change and Conservation Lab, Organismal and Evolutionary Biology Research Program, Faculty of Biological and Environmental Sciences University of Helsinki Helsinki Finland; ^2^ Helsinki Institute of Sustainability Science University of Helsinki Helsinki Finland; ^3^ Finnish Environment Institute Helsinki Finland

**Keywords:** conservation educators, conservation social sciences, environmental education, evaluation, Madagascar, organizations, outcomes, theory of change, ciencias sociales de la conservación, educación ambiental, educadores de la conservación, evaluación, Madagascar, organizaciones, resultados, teoría del cambio

## Abstract

Education is an established tool to enhance human–environment relationships, despite the lack of empirical evidence to support its use. We used theories of change to unpack assumptions about the role of education in conservation. We interviewed practitioners from 15 conservation organizations in Madagascar to typify implicit pathways of change and assess whether emerging pathways echo theoretical advances. Five pathways were drivers of change: increasing knowledge, changing emotional connection and changing traditional cultural practices, fostering leaders, diversifying outcomes, and influencing community and society. These pathways reflect existing sociopsychological theories on learning and behavioral change. Most interviewees’ organizations had a predominant pathway that was often combined with elements from other pathways. Most pathways lacked culturally grounded approaches. Our research reveals assumptions about the role of education in conservation and indicates that organizations had different ideas of how change happens. The diversity of practices reflects the complexity of factors that influence behavior. Whether this diversity is driven by local sociocultural context, interaction with other conservation approaches, or contingencies remains unclear. Yet, typifying the pathways of change and reflecting on them is the first step towards comprehensive evaluation of when and which pathways and interactions to promote.

## Abstract 2

*Article impact statement*: Unpacking conservation education approaches can foster diversification and complementarity of practices, providing foundation for evaluation.

## Abstract (es)

Caracterización de la Visión de los Profesionales de la Conservación sobre el Papel de la Educación

**Resumen:**

La educación es una herramienta consolidada para mejorar las relaciones entre la humanidad y el medio ambiente, a pesar de la falta de evidencia empírica que respalde su uso. Usamos teorías del cambio para desentrañar las suposiciones sobre el papel de la educación en la conservación. Entrevistamos a profesionales de 15 organizaciones de conservación en Madagascar para caracterizar las vías implícitas de cambio y para analizar si las vías emergentes reflejan los avances teóricos. Cinco vías fueron impulsoras del cambio: el incremento del conocimiento, el cambio de la conexión emocional y el cambio de las prácticas culturales tradicionales, el fomento al liderazgo, la diversificación de resultados, y la influencia sobre la comunidad y la sociedad. Estas vías reflejan las teorías socio‐psicológicas existentes sobre el aprendizaje y el cambio de comportamiento. La mayoría de las organizaciones de los entrevistados tuvo una vía predominante que con frecuencia estuvo combinada con los elementos de otras vías. La mayoría de las vías careció de estrategias fundamentadas culturalmente. Nuestra investigación reveló las suposiciones sobre el papel de la educación en la conservación e indica que las organizaciones tenían ideas diferentes sobre cómo ocurren los cambios. La diversidad de prácticas refleja la complejidad de los factores que influyen en el comportamiento. Todavía no está claro si esta diversidad es causada por el contexto sociocultural local, la interacción con otras estrategias de conservación o con las eventualidades. Aun así, caracterizar las vías de cambio y reflexionar sobre ellas es el primer paso hacia la evaluación integral de cuándo y cuáles vías e interacciones se deben promover.

## INTRODUCTION

Education may be a tool for the transformative change needed to protect biodiversity (IPBES, 2019). Environmental education (EE) has been used as a conservation strategy to enhance human–environment relationships (Sauve, 2005) and represents a means to promote proenvironmental attitudes, values, awareness, knowledge, and skills needed to address environmental problems (UNESCO, 1978) and achieve conservation success (Freund et al., 2019). EE spans a range of foundations and discourses. We use EE in a broad sense to encompass learning activities that equip individuals and institutions to respond to environmental challenges and nurture desired individual behaviors and collective actions (Krasny, 2020; Wals & Benavot, 2017). Comprehensive empirical evidence (Ardoin & Heimlich, 2013) is lacking to support the belief that EE benefits conservation. Most EE studies do not document environmental improvement and conservation outcomes (Thomas et al., 2019) and instead focus on easily measured cognitive indicators, such as short‐term changes in knowledge and attitudes (Thomas et al., 2019). This stems from the assumption that we can change behavior by making humans more knowledgeable (Galafassi et al., 2018; Jacobson, 2010). Such approaches align closely with UNESCO's EE guidelines, which endorse a simple linear path from “knowledge to attitudes to behavior” (Krasny, 2020).

A growing body of research suggests a range of dynamic psychological and social factors (e.g., beliefs, social norms, values, emotions, and self‐efficacy) influence human behavior and decision‐making (Marcinkowski & Reid, 2019; West, 2015) (reviewed by St John et al. [2010] and Gifford et al. [2011]). Considering the complexity of human behavior, Krasny (2020) calls for a broader definition of EE that includes nature connectedness, sense of place, identity, norms, social capital, and well‐being. In addition, some researchers criticize the instrumental view of EE as a tool for the environment, rather than an emancipatory tool for social transformation (Sauve, 2014).

It is unclear to what extent the diversity and divergence of theoretical concepts and outcomes are reflected in practice, and questions remain about the ways in which EE can lead to conservation success (Ardoin et al., 2020). Despite calls for a holistic understanding (Johnson, 2013), research is limited in context and audiences, mostly focusing on U.S.‐based systematic reviews (Ardoin et al., 2020; Thomas et al., 2019), and there is almost no documentation of practitioners’ perspectives (but see Ardoin & Heimlich [2013] and West [2015]). To the best of our knowledge, no previous research has focused on practitioners’ perspectives in the Global South.

We sought to unpack the role of education in conservation by typifying the implicit pathways of change that emerge from organizations implementing EE and determining whether emerging pathways echo theoretical advances. The concept of pathways has been used in conservation (Balfour et al., 2019; Biggs et al., 2017) and specifically in EE to define the ways by which EE can contribute to the functioning of the environment, the community, and individuals (Krasny, 2020). We examined the diversity of pathways and their contextualization and identified challenges and potential gaps. We used a qualitative approach to provide insights into the complexity of understanding among EE practitioners and reveal the underlying mechanisms of how EE works as a conservation strategy.

We addressed these topics in a case study of EE programs in Madagascar. Madagascar is a biodiversity hotspot (Myers et al., 2000) and thus the focus of international conservation, international development, and research since the late 1980s (Waeber et al., 2016), and the country has received significant biodiversity funding (Miller et al., 2013). Yet, Madagascar struggles with precipitously declining biodiversity and remains one of the poorest countries in the world, despite its abundant natural resources (Jones et al., 2019). Concerns about biodiversity loss have motivated nongovernmental organizations (NGOs) to include EE in their conservation interventions (Reibelt et al., 2014). However, the extent to which education programs are having an impact and through which pathways is still unclear (Brias‐Guinart et al., 2020).

## METHODS

### Case selection

The Madagascar conservation model parallels that of many other countries in the Global South (Scales, 2014) in which EE is recognized as a tool to address simultaneously conservation challenges and human well‐being. Strategies of EE in the Malagasy school system are weak (Reibelt et al., 2014). Alternatively, such interventions are mostly conducted by international, national, and local NGOs, which mainly target primary school children and youth, but also include a range of community engagement activities (Brias‐Guinart et al., 2020).

We targeted practitioners working in conservation NGOs and other organizations that conduct EE programs throughout Madagascar. We examined nonformal EE activities. Generally, they were not determined by the schools or the standardized school curricula. Rather, they were tailored to the organizations’ needs and interests. However, the lines between formal and nonformal education are fuzzy, and, in some cases, organizations designed their activities to align with the school curricula or conducted their activities in school buildings.

We identified participants via convenience and snowball sampling (Browne, 2005), reaching out first to our contacts from a previous scoping trip (November–December 2018) and then following up with referred participants. We interviewed practitioners from 15 organizations, which covered the wide range of approaches and viewpoints of the most active conservation and education organizations in the country (Appendix S1). We interviewed key informants from each organization, prioritizing those responsible for the education programs. To protect participants’ privacy, they were randomly assigned a letter pseudonym from A to O.

### Data collection

From September through October of 2019, we conducted one‐on‐one interviews with participants that lasted approximately 2 hours each. We interviewed each participant separately because we were interested in understanding the narratives of change (Riessman, 2008) in individual organizations. We asked participants to describe the views of the organization, rather than describing their own beliefs. We did not examine whether their personal opinions were closely aligned with their organization's or whether interviewees were able to separate one from the other. The first author facilitated the interviews in English or French and the audio was recorded.

We adhered to the standard ethical procedures of free prior and informed consent with each participant. The research was approved by the Ethical Board of the University of Helsinki (13 June 2019) and the Ministry of Environment and Sustainable Development, Madagascar (9 July 2019, 182/19/MEDD/SG/DGEF/DGRNE). The positionality and values of the first author—trained in conservation research—influenced the data collection process and subsequent data interpretation.

A theory of change (ToC) is an explanation of how and why an initiative generates a particular change (Belcher & Claus, 2020), and it provides opportunities to reflect critically on one's assumptions (Krasny, 2020). Despite being widely applied in international development organizations, ToC is barely used in the field of conservation (but see Balfour et al., 2019; Biggs et al., 2017). We used ToC as a participatory research tool to identify the pathways that connect education interventions with environmental outcomes (Appendix S2).

At the beginning of each session, the facilitator trained the interviewees in the ToC method to ensure a similar level of understanding (Appendix S3 for protocol details). After that, the facilitator asked 3 main questions to guide the process of drawing the ToC: What is the final goal of your education program? Which intermediate outcomes will lead to your ultimate goal? Which education activities or interventions is your organization conducting?

Each ToC is formed by 2 elements: a diagram representing multiple parallel and intersecting casual pathways and a narrative that provides detail on particular elements.

### Data analyses

**FIGURE 1:**
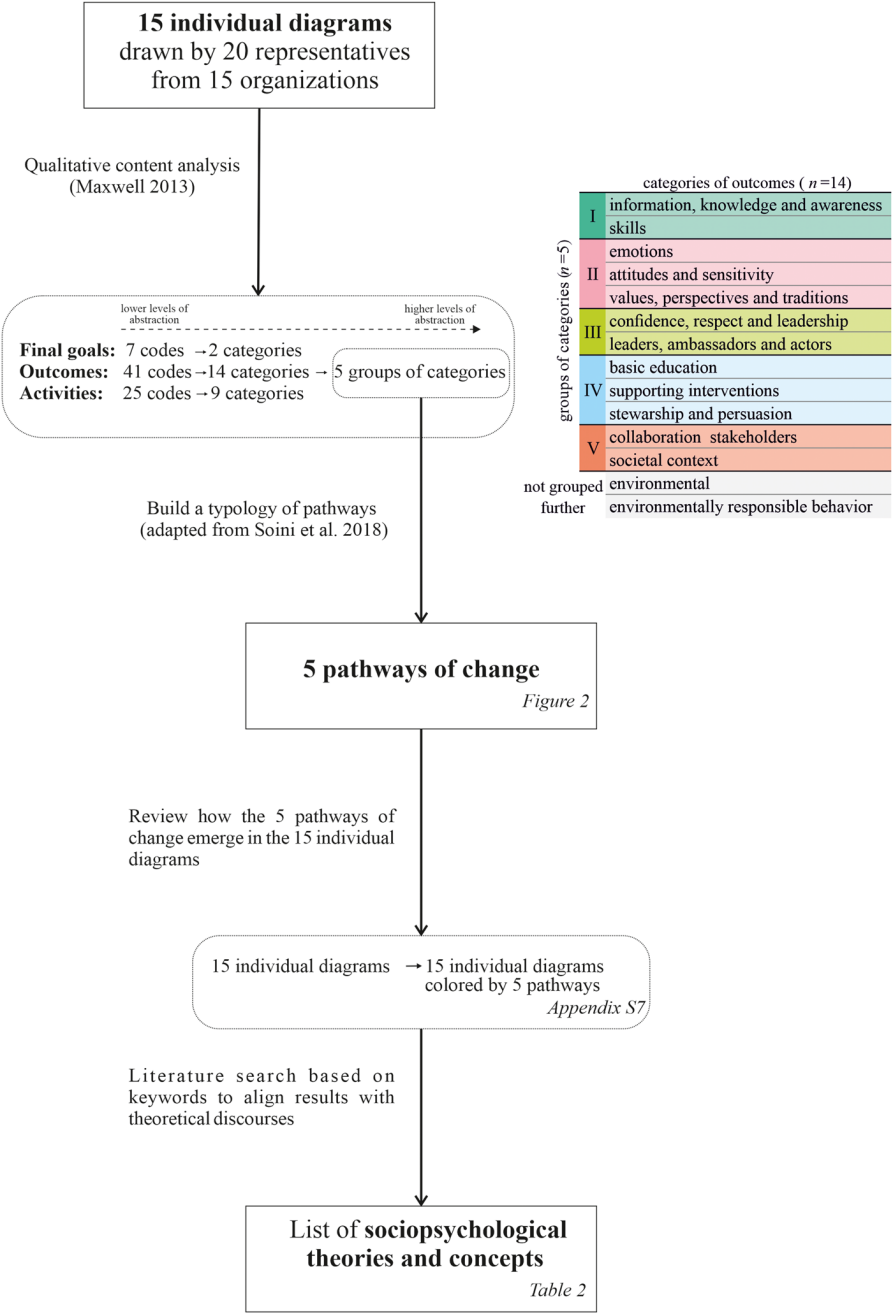
Methodology flow chart. The chart describes each of the steps of data analyses to typify the implicit pathways of change, connecting the steps with the corresponding figures and tables.

We analyzed the data following the steps described in Figure 1. First, we transcribed the interviews and digitized the diagrams, translating them into English. Then, we conducted a qualitative content analysis of the diagrams (Maxwell, 2013) with the software ATLAS.fi 8. This method is a categorizing strategy that involves rearranging the data systematically into categories leading to higher levels of abstraction (i.e., coding). The categories were deductively derived from existing literature (e.g., Ardoin et al., 2015, 2020; Thomas et al., 2019) and inductively generated from the data. We coded the final goals, the outcomes, and the activities separately (Appendices S4 & S5). We focused on outcomes to create a typology of pathways (adapted from Soini et al. [2018]). We built the pathways of change based on the previous categorization (i.e., 5 groups of categories) and the narratives of the interviews to enrich the description of the categorization. Then, we returned to the 15 individual diagrams to identify which pathways were predominant in each of them. Finally, we conducted a literature search to identify the theories and concepts that best aligned with the emerging pathways.

## RESULTS

**FIGURE 2:**
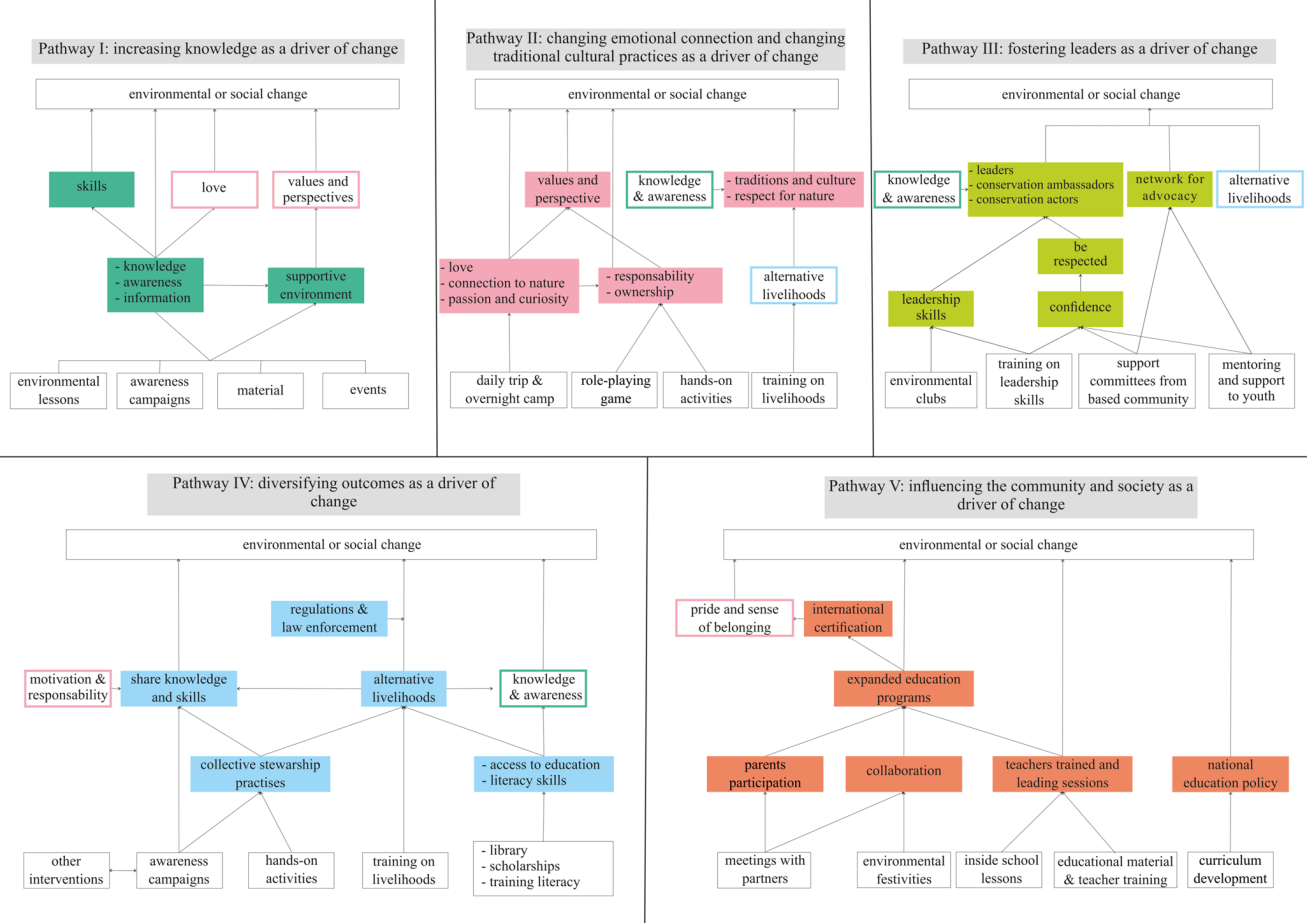
Pathways of change that emerged across the 15 theories of change drawn from interviews with conservation practitioners (bottom row, activities; middle row, outcomes; top row, final goal simplified as environmental or social change; dark green, pathway I; pink, pathway II; light green, pathway III; blue, pathway IV; red, pathway V; solid shading, outcomes that constitute the rationale of the pathway; colored border, supporting elements from other pathways)

Five pathways emerged across the 15 ToCs (Tables 1 & 2). Each pathway was an emerging path that connected the education interventions to the desired final goal and had a series of branching outcomes that illustrated a particular rationale on how change happens. Each of the pathways was connected to elements from other pathways. These elements did not connect the activities to the final goal, but they supported some of the central elements of the pathway. These pathways were idealized, rather than optimal, and can be considered constructs that enhance understanding of the mechanisms on how EE works as a conservation strategy. Thus, Figure 2 provides a visualization of the 5 pathways as drivers of change: pathway I, increasing knowledge; pathway II, changing emotional connection and changing traditional cultural practices; pathway III, fostering leaders; pathway IV, diversifying outcomes; and pathway V, influencing the community and society.

**TABLE 1:** Summary of the characteristics of the pathways of change derived from interviews with practitioners from 15 conservation organizations^a^

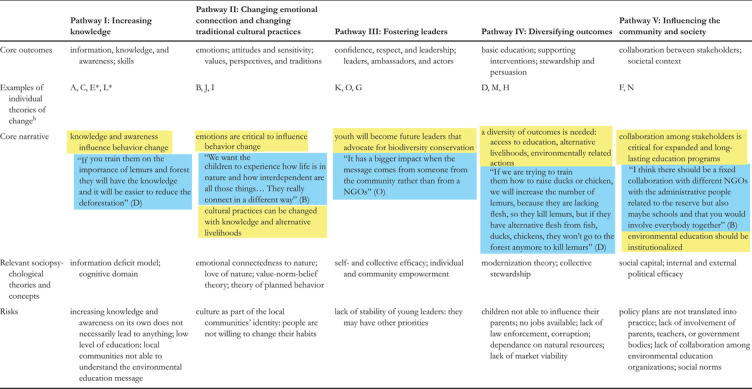

^a^ Letters are interviewees.

^b^ Lack of a predominant pathway in an individual theory of change (*).

**TABLE 2:** Sociopsychological theoretical discourses and models (*) and other factors used to understand human behavior

Concept and Definition	Core pathway	Connection with the pathway
Information deficit model*: oldest and simplest model of proenvironmental behavior. Assumes that increasing environmental knowledge leads to a positive attitude, which in turn leads to proenvironmental behavior (Burgess et al., 1998)	I	Knowledge on its own can lead to a change of attitudes, which will lead to a change of behavior.
Cognitive domain of learning: “what we know,” centered on the acquisition of knowledge and development of intellectual abilities and skills (Bloom et al., 1956)	I	Activities designed to increase knowledge, awareness, and skills.
Emotional connectedness to nature: feeling of being emotionally connected and belonging to the natural world (Frantz & Mayer, 2014)	II	When communities see the forest as a place where they belong, they may begin to develop an emotional responsibility of the care of nature.
Love to nature: children must first learn to love the natural world before they can develop concern for its state or the wish to care for it (Tanner, 1980)	II	Time spend outdoors or in pristine environments can be a core determinant to develop an affection for nature and thus, interest on its conservation.
Value–norm–belief theory*: describes the impact of personal values in determining a personal norm, to act in environmentally protective ways (Stern et al., 1999)	II	Explains the influence of values and beliefs. This theory applies as well to culture, as we understand culture as shared institutions as well as psychological constructs such as beliefs, values, and identifies (Clayton & Myers, 2015; Waylen et al., 2010).
Theory of planned behavior*: attitudes, together with subjective norms and perceived behavioral control, influence behavioral intentions that in turn shape our actions (Ajzen, 1991)	II	Positive attitudes toward nature (integrating values and beliefs) are likely to be linked to proconservation behaviors.
Self‐efficacy: belief in one's ability to succeed in specific situations or accomplish a task (Bandura, 1977)	III	Enhancing confidence and leadership skills can increase individuals’ believe that they can achieve their desired outcomes.
Collective efficacy: group's shared belief in its joint capability to organize and execute the courses of action required to reach goals (Bandura, 1997)	III	Being part of network for advocacy will enhance the group's shared belief of their potential to deliver a change.
Individual and community empowerment: increased control over lives and livelihoods, including control over natural resource management, or increased land‐tenure security (Oldekop et al., 2016)	III	By fostering leaders, this pathway enhances the individual and community capacity and motivation to have agency over natural resources.
Modernization theory: material incentives foster collective concerns and internal motivations for the environment (Inglehart & Welzel, 2005)	IV	People living in nonindustrial or industrializing countries will focus on satisfying immediate needs (e.g., security and economic well‐being), instead of postmaterialist values (e.g., environmental objectives). Under this assumption, a poor and unaware farmer, after receiving material support (such as training on alternative livelihoods), will realize the intrinsic importance of nature.
Collective stewardship: collective conservation‐oriented actions to physically enhance local environments, like planting trees, enhancing wildlife habitat, or participation in wildlife monitoring (Larson et al., 2015)	IV	Communities come together to engage collectively in hands‐on activities and thus become a positive force acting to improve degraded environments.
Social capital: a set of prescriptions, values, and relationships created by individuals in the past that can be drawn on in the present and future to facilitate overcoming social dilemmas (Ostrom & Ahn, 2010)	V	Being able to influence the community will be facilitated by trusting relationships among members of a community, including social connections, trust, and shared social norms.
Internal political efficacy: belief that one understands civic and political affairs and has the competence to participate in civic and political events (Barrett & Brunton‐Smith, 2014)	V	Organizations believe they are able to influence a political change, for example, by participating in curriculum development.
External political efficacy: belief that public and political officials and institutions are responsive to citizens’ needs, actions, requests, and demands (Barrett & Brunton‐Smith, 2014)	V	Organizations are aware of their limited scope, and claim that governmental efforts are essential to institutionalize environmental education at a national level.

Each pathway did not necessarily correspond with all the attributes from a single organization; rather, they reflected elements that were common in most cases. Even though most organizations had a predominant pathway, they often combined supporting elements from other pathways. Most organizations included at least one element from pathway I (93%) and IV (93%) and from pathway II to a lesser extend (67%). Fewer organizations included elements of pathway V (53%) and pathway III (47%) (details in Appendix S6).

### Pathway I increasing knowledge as a driver of change

Pathway I strengthens the role of knowledge as the most important way to achieve behavioral change (Figure 2). The activities were mostly directed to the provision of knowledge. The practitioners stated that the acquisition of knowledge had an effect on the adoption of proenvironmental attitudes, which then linked to the adoption of proenvironmental behaviors: “With the knowledge we can love, and with the love we can protect” (interviewees C and E). Different skills were mentioned by some organisations, from thinking critically, to practical knowledge that provides tools to face environmental problems, to the ability to plant tree nurseries or conduct reforestation. One interviewee emphasized that change rarely happened at the individual level and that it was crucial to foster a supportive environment among the community for people to be able to change their perceptions and behavior.

### Pathway II changing emotional connection and changing traditional cultural practices as a driver of change

This pathway stresses the importance of changing emotional connection to nature and traditional cultural aspects (Figure 2). Interviewees mentioned that a change in emotions could lead to a change in perspectives, from perceiving nature as a resource that can be used to eat or for fun to something that has value in itself that should be protected. Thus, a change in emotions could lead to a change in behavior. Some interviewees stressed the relevance of enhancing the feeling of ownership and responsibility on the protection of biodiversity.

Practitioners frequenly mentioned the importance of direct experiences with nature to foster proenvironment attitudes and emotions. Several interviewees agreed that, even if theoretical knowledge can be transmitted in a classroom environment, emotions develop once the children experience nature directly. Therefore, daily trips or overnight camps in natural areas were considered key to fostering passion and curiosity for flagship species, such as lemurs, and represented an exciting life experience that participants would not forget easily.

Some of the students participated in the student fieldtrip and they absolutely loved it, … A lot of them had never been up a mountain, so that in itself it is a big thing for them. And then when they see this white big lemur, it leaves a real impact (interviewee H).

Some organizations focused on the role of culture or traditional practices, claiming the importance of changing some culturally rooted agricultural practices, such as slash and burn (*tavy*). Training in alternatives to their current livelihoods (pathway IV), such as improving agricultural techniques, was used to motivate people to abandon traditional practices and adopt improved techniques that would prevent the need to expand agricultural land.

There is also a change on the customs, the *fomban drazana*, it means that if the grandmother or the grandfather have done the tavy, they continue to do it, it is the tradition. … The problem is that there is no development training. If they are farmers and they receive training on cultivation, maybe then they will cultivate in another way, or if they know how to do another thing, like handicraft, they can change what they do” (interviewee E).

For some, this change of traditional practices needed to be accompanied by awareness of the importance of the environment (pathway I) to understand the relationship between lemurs, forest, and people and to recover respect for nature.

### Pathway III fostering leaders as a driver of change

This pathway emphasizes the importance of fostering future leaders (Figure 2). Three elements were considered key to reach an environmental impact: having strong leaders, a wider advocacy network, and alternative livelihoods. The interviewees commonly stressed the importance of enhancing leadership skills and knowledge and awareness of environmental issues (pathway I). Likewise, confidence and respect were considered key elements to become a leader, particularly if one is young in a society that values the voices of elders.

After the youth were trained, they were more considered by the communities … Even the mayor of the commune called youth representatives to be present at the commune meeting, which had never happened before (interviewee K).

Interviewees highlighted that it is essential to have a network for advocacy, connecting a wide range of stakeholders, from regional government to private stakeholders. Moreover, the interviewees considered that becoming conservation leaders depended strongly on having alternative livelihoods themselves (pathway IV). Once the leaders adopted alternative livelihoods, such as new agricultural techniques, they would be able to train or influence other members of the community on those alternatives.

Different organizations expressed a range of understanding of leaders and their degree of agency over the management of natural resources. On the one hand, local communities could become ambassadors for conservation when they were involved in the protection of biodiversity as forest guards, guides, or members of the community‐based natural resource management association. On the other hand, local communities were seen as future actors, fully in charge of their natural resources, as one interviewee said:

Not necessarily elders or political leaders, but leaders who feel particularly passionate about an aspect of the environment and are knowledgeable and confident enough to be able to give that information to the community and lead them into managing those resources more sustainably (interviewee G).

### Pathway IV diversifying outcomes as a driver of change

This pathway emphasizes the need of multiple outcomes to have an environmental impact (Figure 2), including access to education, involvement in stewardship practices, sharing among the extended community, and access to alternative livelihoods.

More years of schooling and acquisition of literacy were seen by some organizations as the way to increase understanding of the importance of biodiversity, provide an alternative livelihood, and reduce pressure on the environment.

We have a program that focuses especially in giving scholarships to students, so they can go to school for longer, so they can have a job, rather than fishing, and they can become actors and leaders from their community to make a change in the environment (interviewee O).

The importance of promoting environmentally related actions, such as stewarship practices or sharing knowledge or skills within the community, was commonly mentioned. Collective stewarship practices, such as planting trees or taking care of a vegetable garden, could have a direct impact on the environment by reducing threats to the forest and providing access to alternative livelihoods. Similarly, interviewees highlighted the importance of participants sharing their acquired knowledge or skills (particularly children to parents) until it became normalized, which would be facilitated by participants feeling motivated and having a sense of responsibility (pathway II).

It was frequently stressed that having alternative livelihoods was needed as a path to more sustainable lifestyles.

We propose more sustainable alternatives for them to have an income, so that they don't focus too much on tavy agriculture techniques. For example, in the vegetable garden, we train them how to make compost, they can sell products, and in that way [it] is more sustainable (interviewee M).

The provision of alternative livelihoods was seen as an approach to reduce the dependency of local communities on natural resources (e.g., food, fuel, building materials). In addition, alternative livelihoods could lead to an increased awareness (pathway I) and change in perpection of resources and biodiversity. One interviewee talked about promotion of green enterprises as a path to foster environmental values while solving the problem of youth unemployment. One interviewee mentioned that adoption of sustainable livelihoods should not be exclusively targeted at local communities, but also to other relevant actors, such as companies or the government. Relatedly, for some organizations, the adoption of alternative livelihoods was linked to compliance with regulations and law enforcement, as well as respect of local customs and social norms (known as *DINA*).

### Pathway V influencing the community and society as a driver of change

This pathway enables change at the community and societal level, highlighting the role of collaboration among stakeholders and the importance of institutionalizing EE (Figure 2).

The interviewees commonly emphasized that collaboration among stakeholders at the community level was essential, ranging from individuals, such as nature guides, to local researchers, to parents, up to local governmental representatives, law enforcement agents, and the private sector. Likewise, several organizations worked within the formal school setting, particularly primary schools. This collaboration was key to extending the implementation of education programs on a bigger scale, which was usually beyond the options available to individual organizations that often target single school classes or specific groups of students. Along these lines, teacher training was done to reinforce knowledge and competence and as an investment to extend EE.

At the moment the activities are done in pairs: one teacher and one animator. But the idea is that is the same teacher that can do that alone. So each school break, we organize training for the teachers to strenghten their competence and capacities, and we hope that in the near future they can do that [the teaching] alone (interviewee O).

Organizations often highlighted the need to institutionalize EE, recognizing the importance of the insertion of EE (or education for sustainable development for some interviewees) in the national school curriculum. Others mentioned nongovermental means, such as the use of international certifications for green school programs. These approaches integrated the direct environment of the school in the learning process and aimed to change values within the entire educational community so as to expand to the wider community

When the school is certified they will acquire new values, and then, when they have the values, they will have the pride and sense of belonging to the school (interviewee N).

## DISCUSSION

### Diversity of pathways and interactions with sociopsychological concepts

Our results highlight that the conservation organizations in our study have different rationales on how change happens, which aligns with sociopsychological theories on learning and behavioral change. Environmental knowledge is critical for making informed decisions and for fostering an environmentally literate society. The prevalence of knowledge was equally reflected in our pathways. For a long time, EE practitioners have focused on fostering knowledge or attitudes (Freund et al., 2019; Jacobson, 2010), assuming a linear path to behavioral change; that is, “if people know enough, they'll change. Or if they feel in a certain way, they'll act differently” (Heimlich, 2010, p. 184). This assumption, based on the information deficit model (Burgess et al., 1998), was held by some interviewees, who conveyed that increasing environmental knowledge would lead to a positive attitude toward the environment (e.g., love) that would lead to proenvironmental behaviors (pathway I). Similarly, this assumption was held by interviewees who run projects involving contact with nature. They explained that if participants develop affection for nature (linked to the concept of emotional connectedness to nature [Frantz & Mayer, 2014]), it would lead to increased proenvironmental behavior (pathway II).

These results suggest that—to a certain extent—EE organizations are following the UNESCO guidelines to frame their interventions. The rationales in pathway I and II are closely aligned with the Tbilisi Declaration of 1977, which can be simplified as a knowledge–attitudes–behavior theory of change (Krasny, 2020): “EE activities create the knowledge, skills, and awareness needed to address environmental challenges and foster attitudes, motivations, and commitments, which lead audiences to make informed decisions and to take action” (UNESCO, 1978 [as cited in Krasny, 2020, p. 8]). Part of the interviewees remained strongly connected with this traditional thinking on EE, even though research shows that moving people to action is difficult and that there is not a specific factor sufficient to lead to a specific behavior (Kollmuss & Agyeman, 2002). Instead, theoretical advances call for a dynamic and complex net of components that influence behavior (Krasny, 2020; Marcinkowski & Reid, 2019). This complexity is reflected in our results as well. Most individual ToCs combined elements from different pathways, and all interviewed organizations but one included at least one outcome from pathway IV (Appendix S6). This may suggest that practitioners are aware of the limitations of the traditional approach to EE and have adopted a much broader understanding of EE that includes a diversity of outcomes that nurture change from the individual to the societal levels.

Since the Tbilisi Declaration, EE has undergone much change, such as the emphasis on social justice, community and youth development, and empowerment (Freund et al., 2019; Krasny, 2020). Our results further support this approach. Several interviewees highlighted the empowerment of local communities to manage and use their natural resources (pathway III). This aligns with calls for an inclusive model of conservation in which local communities have agency over their natural resources, in contrast with the colonial legacy of fortress conservation (Corson, 2017; Scales, 2014). Similarly, several researchers claim the need to strengthen tenure rights as one of the urgent actions to address the decline of biodiversity (Jones et al., 2019).

Yet, can children or youth be agents of change? In Western societies children engage in proenvironmental behavior, particularly in private spheres such as recycling (Clayton & Myers, 2015), and may influence adults (Zampos, 2011). However, the potential of youth to have an impact on adult agendas is uncertain (Porter et al., 2012), particularly in societies in which young people's agency is partly limited by age hierarchies (Smith, 2013). The traditional social Malagasy system is hierarchical. Ancestors are the most powerful, followed by the elders of the community (Reibelt et al., 2017), even if their worldviews are increasingly influenced by migration, modernization, and demographic change (Golden & Comaroff, 2015). Under this system, young people rarely question older people or traditional practices (Reibelt et al., 2017). Therefore, fostering environmental leaders is strongly connected with strengthening youth's sense of empowerment, efficacy, and social capital (Table 2) and involves a series of key elements to influence social development: leadership, trust, and ability to collaborate (Jacobson et al., 2006). Some of these elements are mentioned in pathway III. Hands‐on activities and stewardship practices (pathway IV) are examples of strategies that foster self‐efficacy (Beaumont, 2010). They allow youth to master a skill or behavior, to learn from inspiring community members, and to develop a sense of community, all of which build trust and the ability to collaborate.

At the same time, hands‐on activities improve the skills and expertise of the participants, which can improve their economic situation and thus increase their sense of empowerment (Pohnan et al., 2015). Livelihoods programs were an integral part of many of the organizations interviewed (pathway IV), supporting the idea that financial concerns may be a strong predictor of environmental behaviors (Freund et al., 2019). Many of the organizations included training in agricultural techniques as part of their EE. This aligns with the argument that the main drivers of environmental degradation have been widespread poverty, increasing population, and absence of resources and techniques to improve the productivity of agricultural land (Sussman et al., 1994). This narrative, reminiscent of the modernization theory of Inglehart and Welzel (2005), is common in projects based on the assumption that raising income in rural areas will foster more collective concerns, solving the problem of environmental degradation (Chambers et al., 2020). However, even if most conservation organizations tackle local drivers of biodiversity loss (St John, 2010), we believe it is important not to oversimplify and to be aware of the complexity of drivers of environmental change, such as the influence of external drivers and powerful elites (Scales, 2014). For example, rapidly changing threats (e.g., escalating mining pressures) are increasingly linked to organized crime and involve people far from local areas (Cabeza et al., 2019; Scales, 2014).

The behavior of an individual is woven into a complex web of personal, social, and cultural context (Dickman et al., 2013). Thus, to have a significant effect, change must occur beyond the individual level (Clayton & Myers, 2015). Pathway V highlights this by referring to the collaboration among various agents of society: members of local communities and governmental and NGOs.

Yet, the pathways we delineate are idealized; thus, there are several barriers that would prevent their fruition. Most interviewees mentioned risks (Table 1) related to shifting the focus of EE from the individual to the community level, such as the involvement of teachers, parents, and governmental bodies. One of the limiting factors may be social norms—the standards of behaviors that society expects (Krasny, 2020). For instance, parents’ participation in school activities, such as alternative agriculture techniques, may not trigger a change in behavior in their agricultural practices because they may be hindered by social norms (e.g., the practice of tavy following their ancestors’ traditions). Therefore, it is essential to build social capital among the community (Ostrom & Ahn, 2010) and a sense of collective efficacy (Bandura, 1997) to enable collective behavior. Again, engaging participants in collective hands‐on activities, such as stewardship practices involving intergenerational participation and cooperation with civil society organizations, fosters trust and social connections (Krasny, 2020) and builds a sense of self‐ and collective efficacy and empowerment to take on more challenging behaviors (Lauren et al., 2016).

In addition to these sociopsychological factors, environmental decisions are influenced by demographic factors (e.g., years of schooling) and “external factors,” such as geophysical elements (e.g., accessibility of a resource), the regulatory context (e.g., policies and rules), and technological elements (e.g., tools available) (Gifford et al., 2011). Interviewees recognized these contextual barriers, mentioning the low levels of education, market viability of alternative livelihoods, law enforcement, and policy plans. At the same time, EE interventions incorporated some of these external elements, for example, by aiming to influence national educational policy (pathway V) and access to education (pathway IV).

### Importance of sociocultural context in EE for conservation

The “internal factors” of emerging pathways have different effects in different sociocultural contexts (Manfredo et al., 2020). Thus, the success of conservation interventions may depend on the extent to which those educational projects are tailored to the local cultural context (Waylen et al., 2010). This is particularly relevant in Madagascar, where the environmental agenda has long been influenced by international agents (Mercier, 2006; Waeber et al., 2016) whose perspectives may differ from those of Malagasy communities (Reibelt et al., 2014). Thus, researchers emphasize the need to design EE programs that consider local community needs, values, and knowledge systems (Brias‐Guinart et al., 2020; Reibelt et al., 2014).

The pathways discussed above may not necessarily be tailored to the sociocultural contexts. For instance, the potential of youth to influence adults in a Malagasy society or the clash with institutional barriers, such as the persistent model of fortress conservation when fostering local leaders. Another example is the prevalence of knowledge in the pathways of change, *knowledge* always referred to *scientific knowledge*, and none of the interviewees explicitly mentioned that other knowledge systems were supported in the education interventions, even if some recognized the existence of local knowledge. As explained by interviewee B:

Those children living around the forest, they know everything, like what the forest looks like, what kind of animal is that. They have seen all lemurs. … They have been growing up with them… So they know a lot of things, but they don't know the relationship. They use them as a resource, to eat, or for fun. And this [environmental] awareness is not there at all.

Despite international calls for recognition of the diversity of values and knowledge systems (Díaz et al., 2019), our results suggest no extensive changes have happened on this front in Madagascar. Our findings are consistent with those of Korhonen and Lappalainen (2004), who discussed the lack of local knowledge in EE programs in Madagascar. A small number of interviewees mentioned that cultural or traditional practices should be changed to further conservation goals (pathway II). With this, those conservation actors were undervaluing deeply embedded socioecological practices such as tavy as “simple environmental practices” without accounting for the effects that the adoption of new agricultural techniques would have on local social life and custom (Desbureaux & Brimont, 2015).

This finding may be tentative, considering the methods we used. We did not inquire specifically about the relevance of the sociocultural context. We are aware that some organizations have made outstanding advances on this front. For example, Reibelt et al. (2018) present a role‐playing game developed by Madagascar Wildlife Conservation. This game is an educational tool created with a participatory approach that enables a diversity of value systems, worldviews, and aspirations. Questions remain to what extent culturally grounded approaches to EE in the context of biodiversity conservation are extensively implemented in Madagascar.

### Implications for designing and evaluating future transformative EE interventions

Our results contribute to the understanding of the role of education in conservation, delineating some disconnects between theory and practice. Many of the practitioners based their practices, deliberately or not, on the UNESCO guidelines, which is reflected in the prevalence of knowledge, awareness, attitudes, and skills in our results. This trend may be explained by a lack of accessibility to research results and a lack of guidelines to connect theory to practice that would reflect the theoretical advances in the factors influencing behavior. Still, we are aware that practitioners often lack the time and resources to carefully design their education programs according to the scientific evidence.

At the same time, our results reflect calls to broaden understanding of EE (Krasny, 2020) and to consider EE as a wider cultural and social force, including the empowerment of local communities and activities to build efficacy, trust, and connections. Thus, although most theoretical advances have focused on single factors, it is key for future research to embrace the complexity of the sociocultural context where education programs take place when implemented as conservation tools in the Global South. Similarly, a deeper understanding is needed as to the practical obstacles of putting theory into practice and how these obstacles can be accounted for in the development of theory.

The diversity of pathways in our results supports similar findings that suggest there is no single way to foster environmental awareness, concern, or activism (Reibelt et al., 2017), but rather a range of pathways that need to be complemented to reach the final goal. Likewise, our results emphasize that EE approaches need to be accompanied by other structural solutions, such as access to alternative livelihoods and policy changes. The diversity of pathways may indicate that there is no one‐size‐fits‐all approach in EE, but it may also just reflect different schools of thoughts and not necessarily match the needs or effectiveness of the programs. Yet, typifying the pathways of change and reflecting on them is the first step toward identifying suitable outcomes for a more comprehensive evaluation of when and which pathways and interactions to promote. Many of the interviewees recognized it was their first time reflecting on the linkages between activities, outcomes, and impacts and that they were unaware of what the ToCs were behind their education programs. This manifests the context of EE in Madagascar, and probably elsewhere in the Global South: many conservation organizations are implementing EE as one of their tools to achieve conservation success, with good intent. Unfortunately, education programs are usually underfunded and are led by biologists without pedagogical training. Thus, educational approaches are often just copied from other projects without efficacy evidence or context‐dependent design. This is a potential waste of resources (financial, technical, and human) and could backfire in resource‐dependent communities. Thus, by examining practitioners’ perspectives, our results contribute to understanding of the assumptions of how a system works, which is key to designing transformative interventions (Galafassi et al., 2018).

By unpacking the role of education in conservation, our results have implications for future practice. Our results suggest that top‐down conservation approaches are still prevalent and that many organizations are exclusively promoting Western values instead of recognizing local knowledge, needs, and values (Scales, 2014). We emphasize, contrastingly, the importance of localizing EE and acknowledging a diversity of worldviews, including local conceptions of nature that diverge from Western epistemologies. We echo the calls of previous researchers that propose an approach to EE through which individuals can learn to address complex social issues through participatory, inclusive, and emancipatory learning approaches that incorporate a political dimension to EE to promote active citizenship (Sauve, 2014). If EE is to continue to be a foundational aspect of many conservation initiatives, it should equally be a strategy to recognize, support, and celebrate the diversity of cultural and knowledge systems and to enhance the process of participation in the decision‐making of local communities on natural resource management.

## Supporting information

Appendix S1. Descriptive analysis: profile of conservation organizations interviewed
Appendix S2. Concept of theory of change
Appendix S3. Interview protocol
Appendix S4. Descriptive analysis of final goals, outcomes and activities
Appendix S5. Codebook of intermediate outcomes: category, code and explanation
Appendix S6. Diversity of typified pathways in individual ToCs
Appendix S7. Individual ToC diagrams colored into the 5 pathways
