# Supplementary material for: Typifying conservation practitioners’ views on the role of education
Source: Conserv Biol. 2022 Feb 21;36(4):e13893. doi: 10.1111/cobi.13893 (PMC9543612; doi:10.1111/cobi.13893)
Supplement: Supplementary file 1 — Appendix S1. Descriptive analysis: profile of conservation organizations interviewed Appendix S2. Concept of theory of change Appendix S3. Interview protocol Appendix S4. Descriptive analysis of final goals, outcomes and activities Appendix S5. Codebook of intermediate outcomes: category, code and explanation Appendix S6. Diversity of typified pathways in individual ToCs Appendix S7. Individual ToC diagrams colored into the 5 pathways [file COBI-36-0-s001.docx]

# Supporting information

## Appendix S1. Descriptive analysis: profile of conservation organizations interviewed

Table S1. Descriptive characteristics of the interviewees. Including: i. alias, ii. role of the practitioner interviewed, iii. identity of the organization, iv. geographical scope: local (works in a single community/area), regional (works in a single region), national (works in different regions/all around Madagascar), v. organizational size refers to number of employees: micro (less than 20), small (less than 100), medium (100-200), large (more than 1000), vi. starting year of the education intervention described during the interview.

## Appendix S2. Concept of theory of change


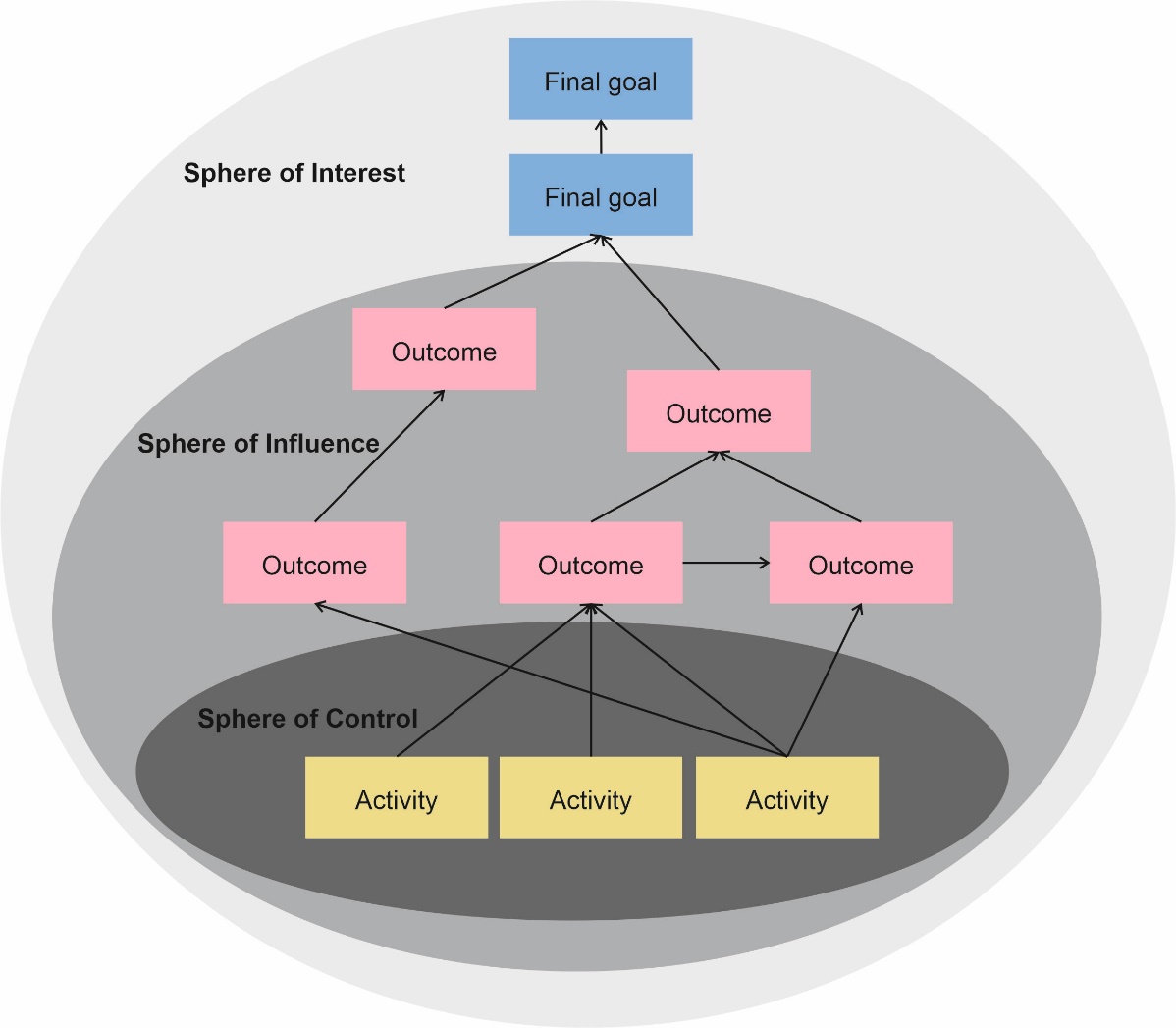


## Figure S2. Concept of the theory of change (ToC) (Adapted from Belcher & Claus, 2020). ToC uses a backwards mapping process: first, the desired final goals are identified (at the top in dark blue); second, all the earliest changes that need to occur to reach the final goal (outcomes, in the middle in pink); and third, the interventions that the organization carries out to achieve those changes (activities, at the bottom in light yellow). Finally, each intervention is linked to one or more outcomes, creating a complex web of assumptions about what needs to happen to bring about change (Center for Theory of Change, 2021).

## Appendix S3. Interview protocol

The protocol in this research was inspired by the methodology detailed by LaMere et al. (2020). Before the interviews, we conducted various pilot sessions with five Malagasy researchers and practitioners, and revised accordingly the research protocol and the guiding questions.

**Participant learning.** At the beginning of each elicitation session, the facilitator prepared the participants for the task of representing their views as a theory of change, by explaining what it meant and how to interpret and visualize theories of change, using a simplified example from the theory of change developed by Balfour et al. (2019). At the time, we believed that that theory of change to grow numbers of African rhino at a conservation site was general and vague enough to avoid the anchoring effect (Oppenheimer et al., 2008). As this method was new for most of the interviewees, the explanation was done as thorough as possible to ensure that the participant would not feel uncertain or uncomfortable with the process of drawing the theory of change (LaMere et al., 2020).

**Direct elicitation.** The diagrams of the theory of change (ToC) were hand-drawn on large sheets of paper and participants chose to either draw the diagrams themselves or allow the facilitator to draw. When the facilitator was responsible for drawing, she encouraged the interviewees to describe explicitly the relationships between outcomes, constantly reminding them to interject if the theory of change was not drawn according to their beliefs, and regularly confirmed the theory of change’s accuracy.

After defining the final goal, the outcomes and the activities, participants were asked to draw the arrows to connect the different variables. Drawing these pathways was the last step in the elicitation process, to avoid disrupting the stakeholder’s flow of thoughts as they constructed their theories.

During the elicitation session, the facilitator asked the stakeholder three primary questions to guide the process of drawing the theory of change, and to identify the final goal(s), the outcomes and the activities:

- What is the final goal of your education program?
- Which intermediate outcomes will lead to your ultimate goal?
- Which education activities/interventions is your organization conduction?

These questions were reiterated as needed over the course of the elicitation session until reaching saturation. Other questions asked by the facilitator were meant to provide clarification, keep the session on task or prompt discussion while influencing the participants as minimally as possible.

## Appendix S4. Descriptive analysis of final goals, outcomes and activities

Interviewees held different views on what is the final goal of their interventions. The goals reported by the interviewees converged into seven distinguishable codes that were further grouped in two categories: either environmental or social change (Figure S3A). For example, while some mentioned that the overall goal was to improve the habitat or species conservation, others focused on social impacts, that ranged from increased awareness, to behavior change, promoting sustainable life and empowering local communities to become conservation actors. Most organizations had a single final goal, with slightly larger proportion describing a social rather than an environmental goal. Few organizations mentioned both: a social final goal, e.g. “Local communities are aware of the importance of the environment ”, followed by an environmental final goal, such as “healthy environment” (interviewee J).


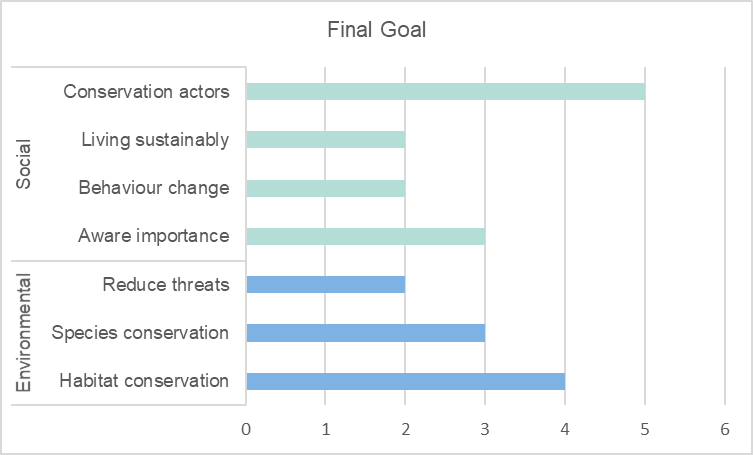


Figure S4A. Categories of impacts: social impacts (in light blue) and environmental impacts (in dark blue).

We identified a larger number of codes for outcomes (41). We grouped them gradually into higher levels of abstraction (see Table S2), converging into fourteen different categories of outcomes, which ranged from cognitive, behavioral, community, societal and environmental outcomes (Figure S3B). The most popular outcomes were cognitive and community, followed by behavioral, and finally societal and environmental ones. The environmental related outcomes (related to species and habitat conservation, and threats reduction) and the behavioral outcomes (e.g. environmental responsible behavior) were not considered for the definition of pathways. They were considered as part of the final goals instead.


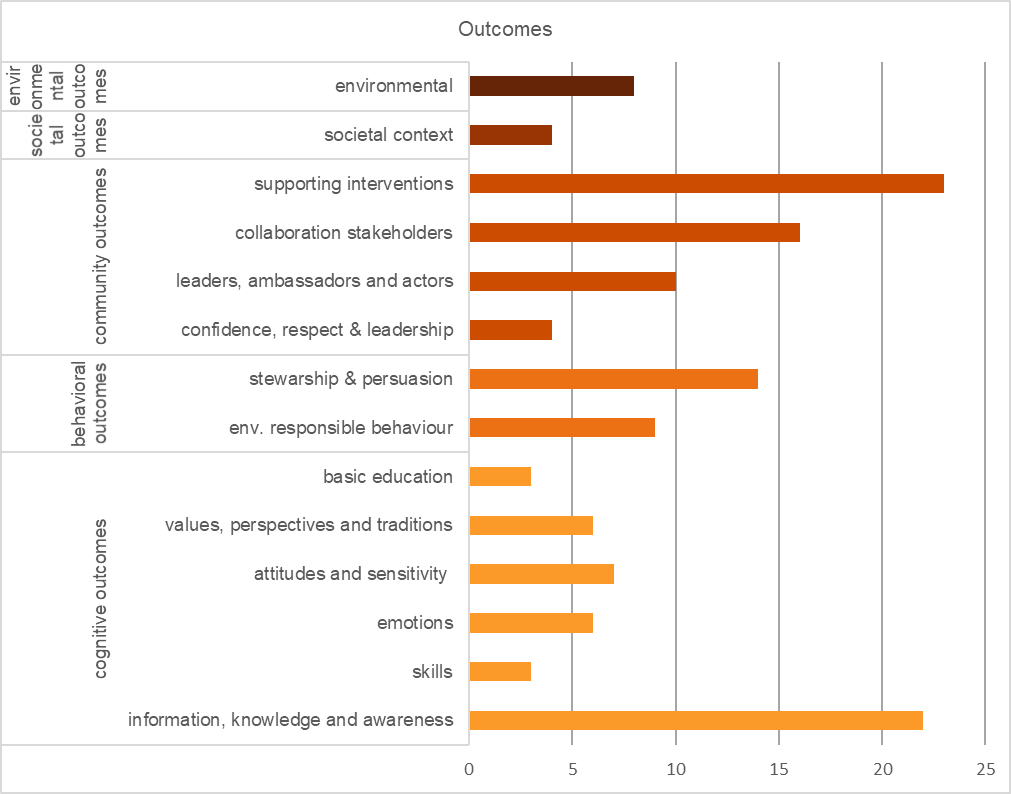


Figure S4B. Categories of outcomes divided in 4 different groups (from light to dark grey): individual, community, societal and environmental level.

The activities reported by the participants converged into different codes (25) which we further classified into 9 emerging categories, illustrating a wide range of pedagogical approaches (Figure S3C) (from more to less common): supporting interventions, one-off interventions, provision of material, environmental lessons for children and youth, villagers training, mentoring and training on leadership, hands-on activities, fieldtrips, and teachers training. On average, programs had 9 outcomes (ranging from 4 to 15), and 7 activities (from 4 to 13).

##
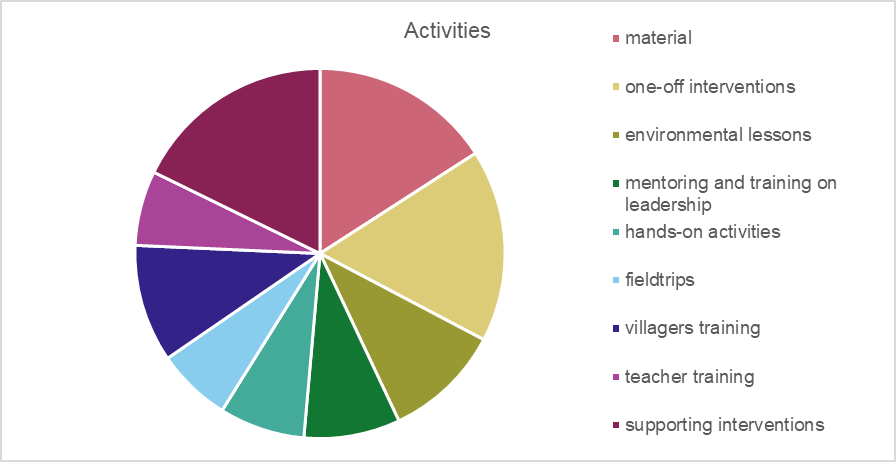


Figure S4C. Categories of activities.

## Appendix S5. Codebook of intermediate outcomes: category, code and explanation

## Appendix S6. Diversity of typified pathways in individual ToCs

|  |  |  | ideal-type pathways | | | | |
| --- | --- | --- | --- | --- | --- | --- | --- |
|  |  |  | **I** | **II** | **III** | **IV** | **V** |
|  | individual ToCs | A | X |  |  | X |  |
|  |  | B | X | X |  | X | X |
|  |  | C | X | X |  | X |  |
|  |  | D | X | X |  | X |  |
|  |  | E | X | X |  | X | X |
|  |  | F |  |  | X |  | X |
|  |  | G | X | X | X | X |  |
|  |  | H | X | X | X | X |  |
|  |  | I | X | X |  | X | X |
|  |  | J | X | X |  | X |  |
|  |  | K | X |  | X | X |  |
|  |  | L | X |  | X | X | X |
|  |  | M | X | X | X | X | X |
|  |  | N | X | X |  | X | X |
|  |  | O | X |  | X | X | X |
| num. of individual ToCs | |  | 14 | 10 | 7 | 14 | 8 |
| % | |  | 93% | 67% | 47% | 93% | 53% |

Table S6. Diversity of typified pathways (x axis) in each individual ToC (y axis). Grey cells marked with X show the predominant pathway for that individual ToC, and white cell marked with X shows a pathway that supports that ToCs but is not predominant.

## Appendix S7. Individual ToC diagrams colored into the five pathways


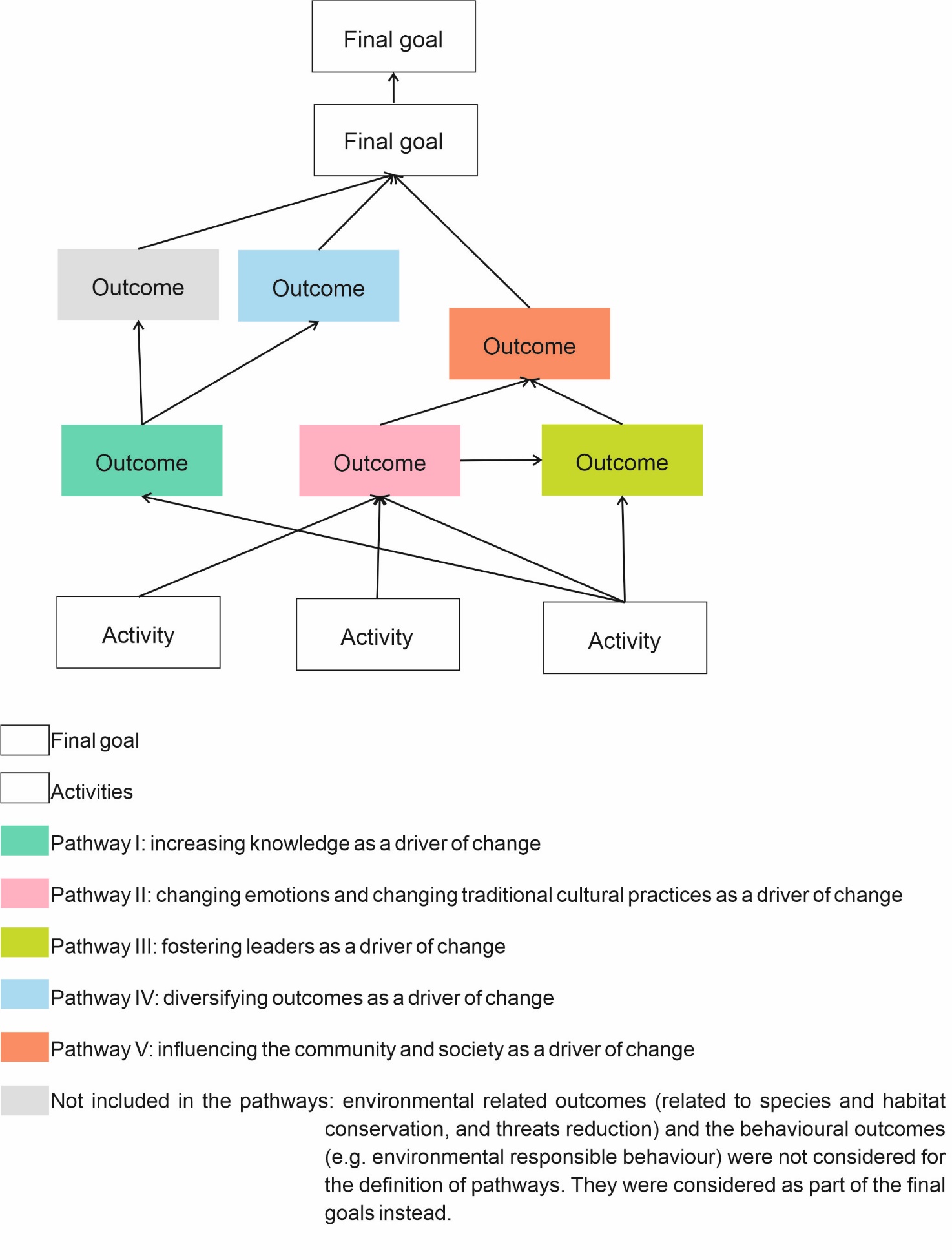
Figure S7W. Legend to read the individual ToCs. The individual ToCs presented are simplifications of the original ones. Proper nouns of places or education material have been removed to protect the participants’ privacy.

**
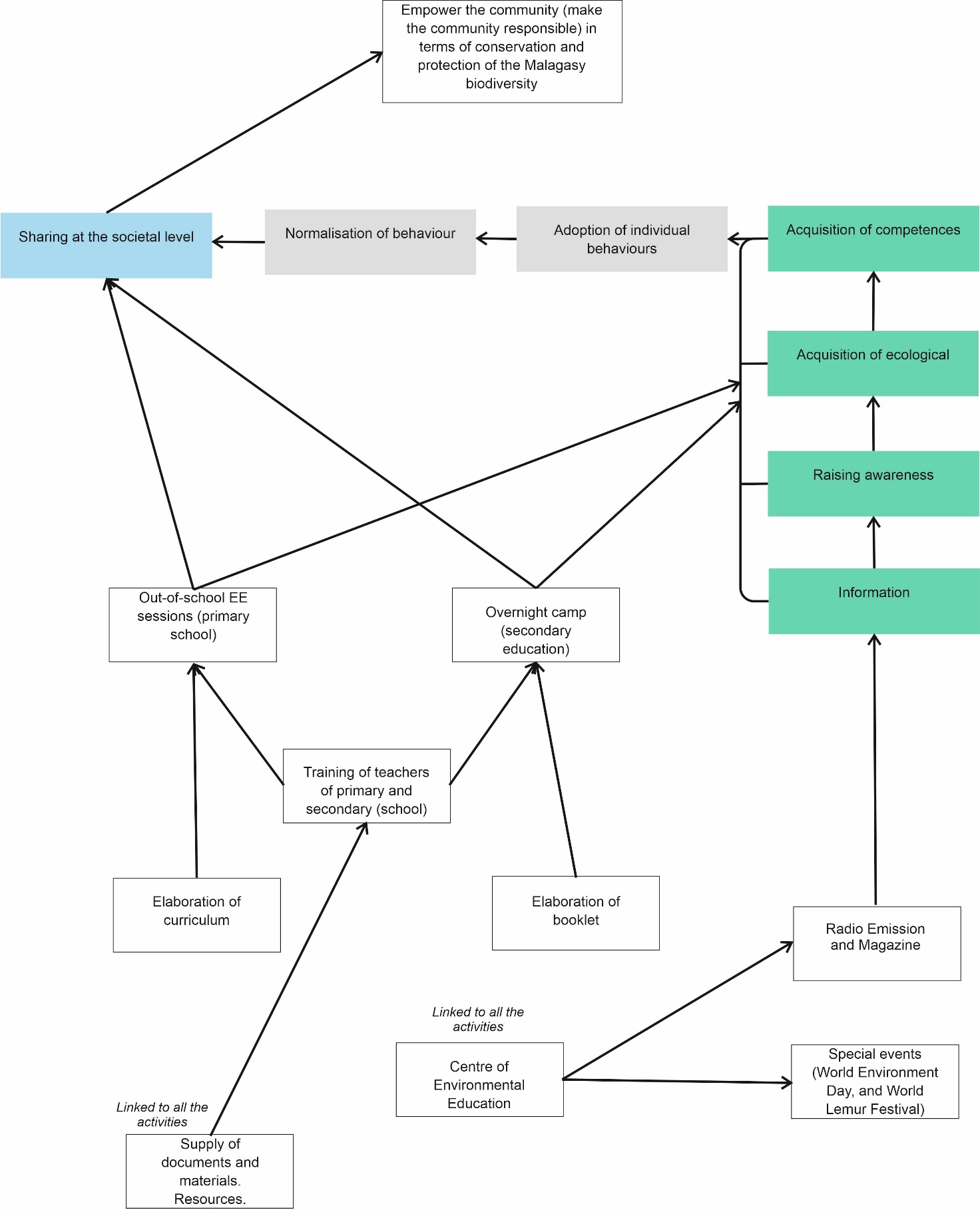
**

Figure S7A. Individual ToC interviewee A


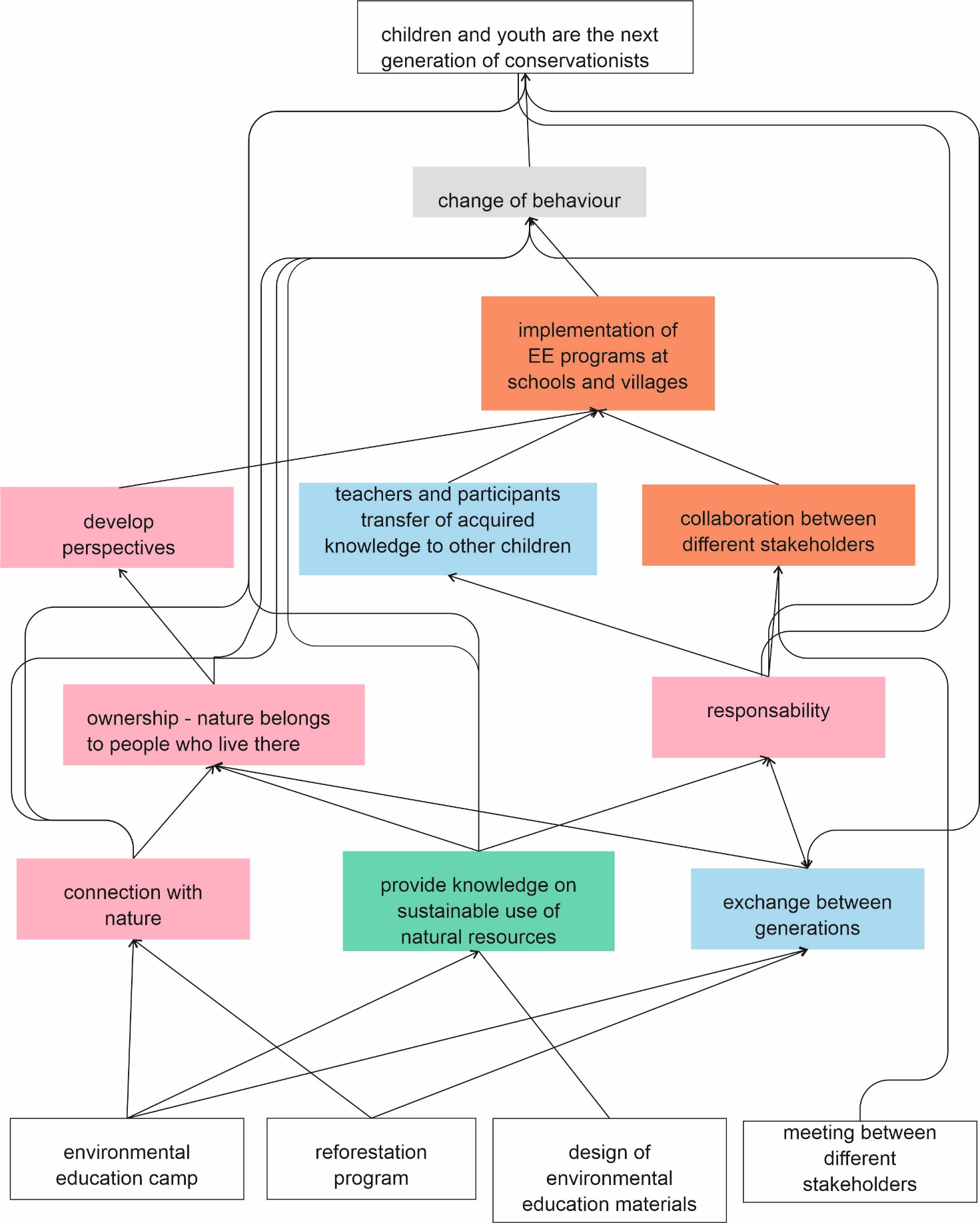


Figure S7B. Individual ToC interviewee B


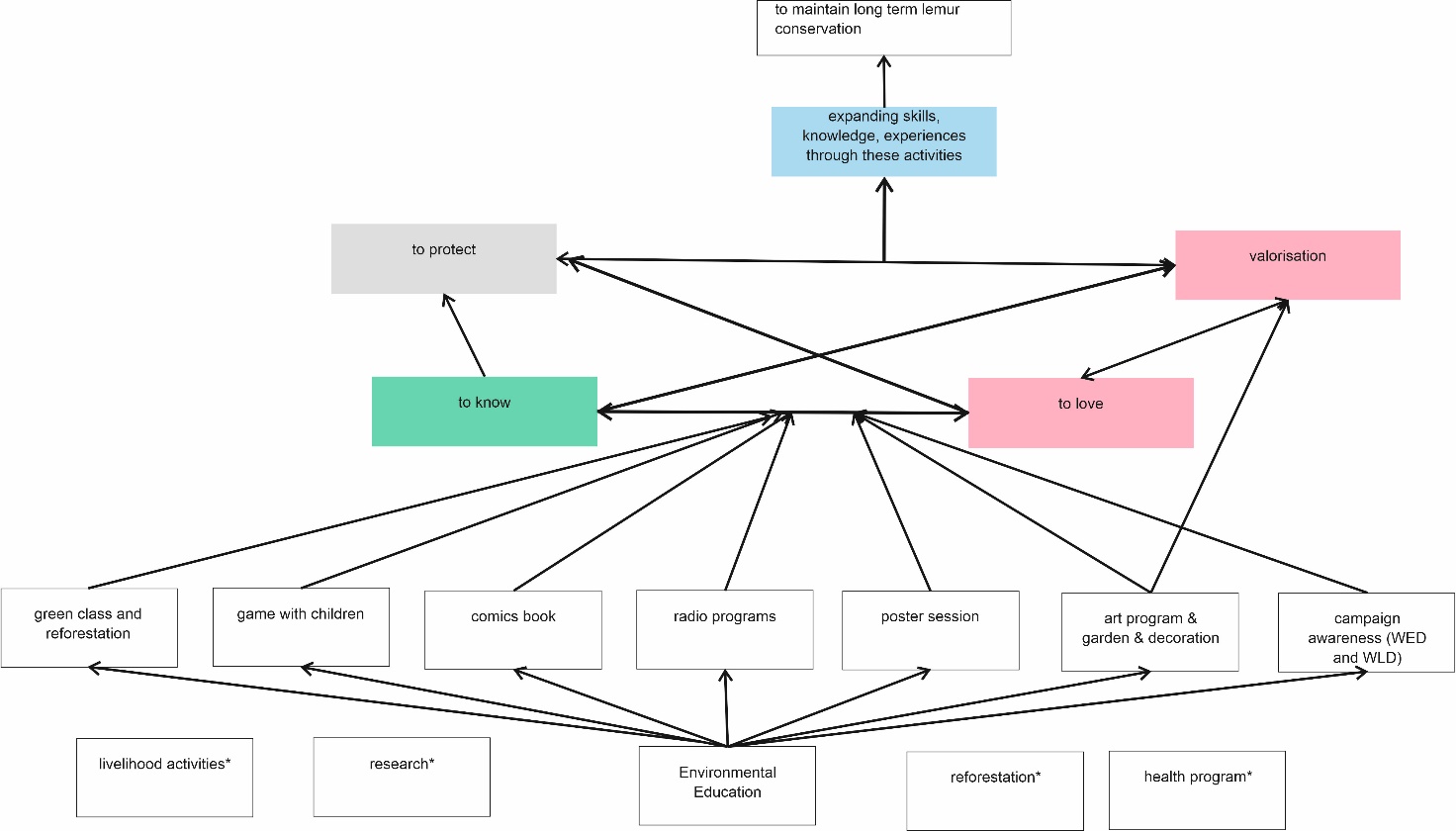


Figure S7C. Individual ToC interviewee C


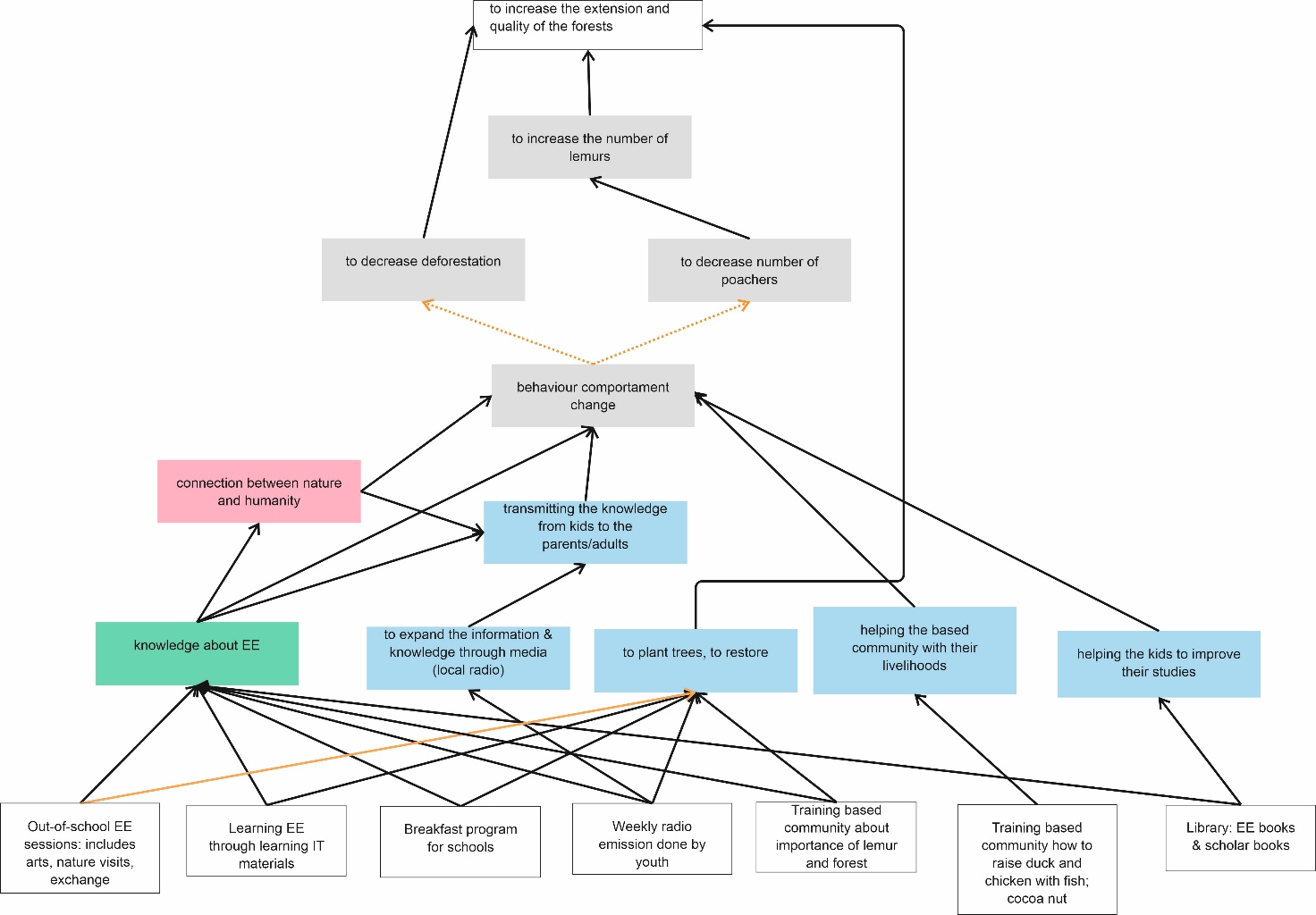


Figure S7D. Individual ToC interviewee D


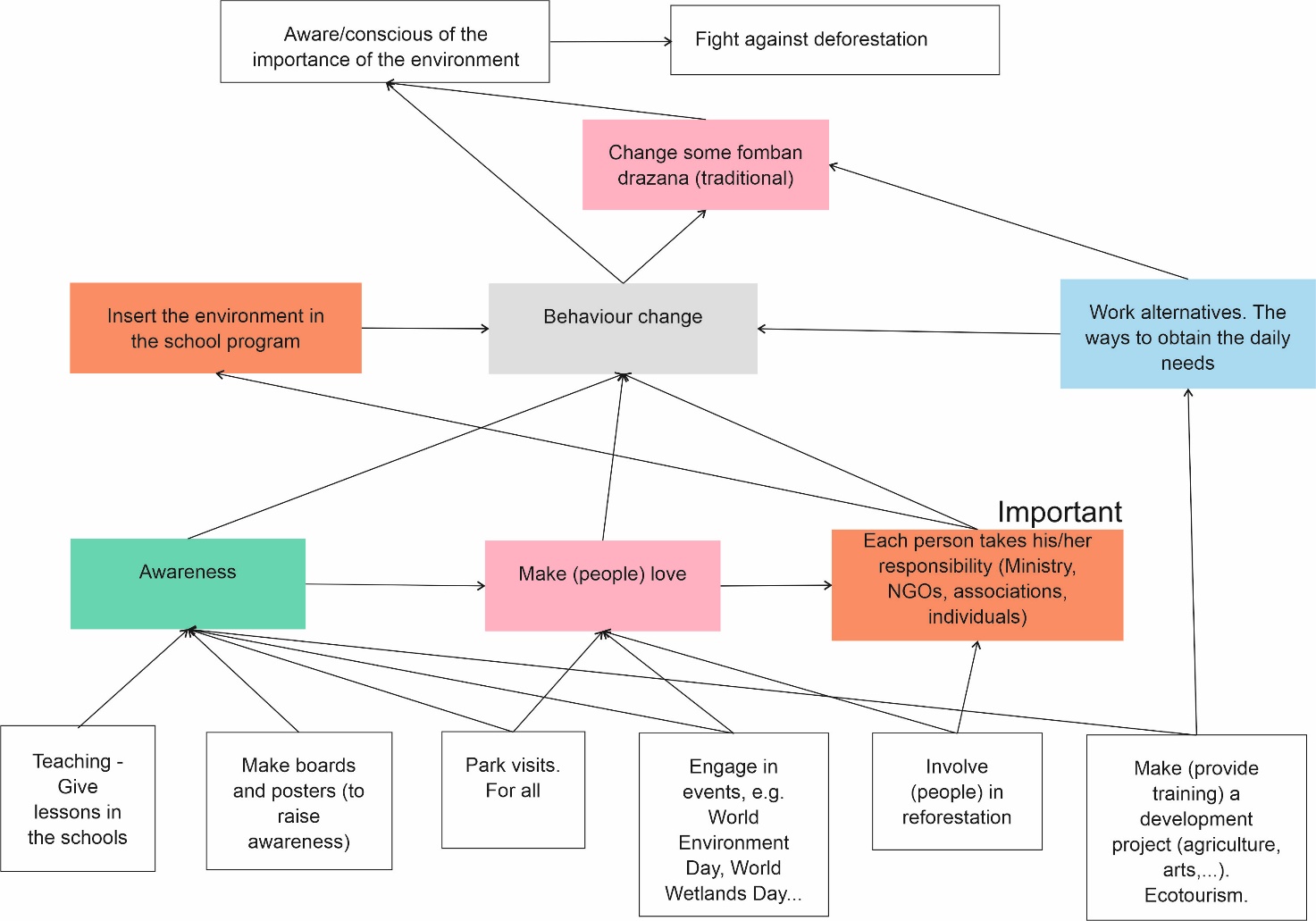


Figure S7E. Individual ToC interviewee E


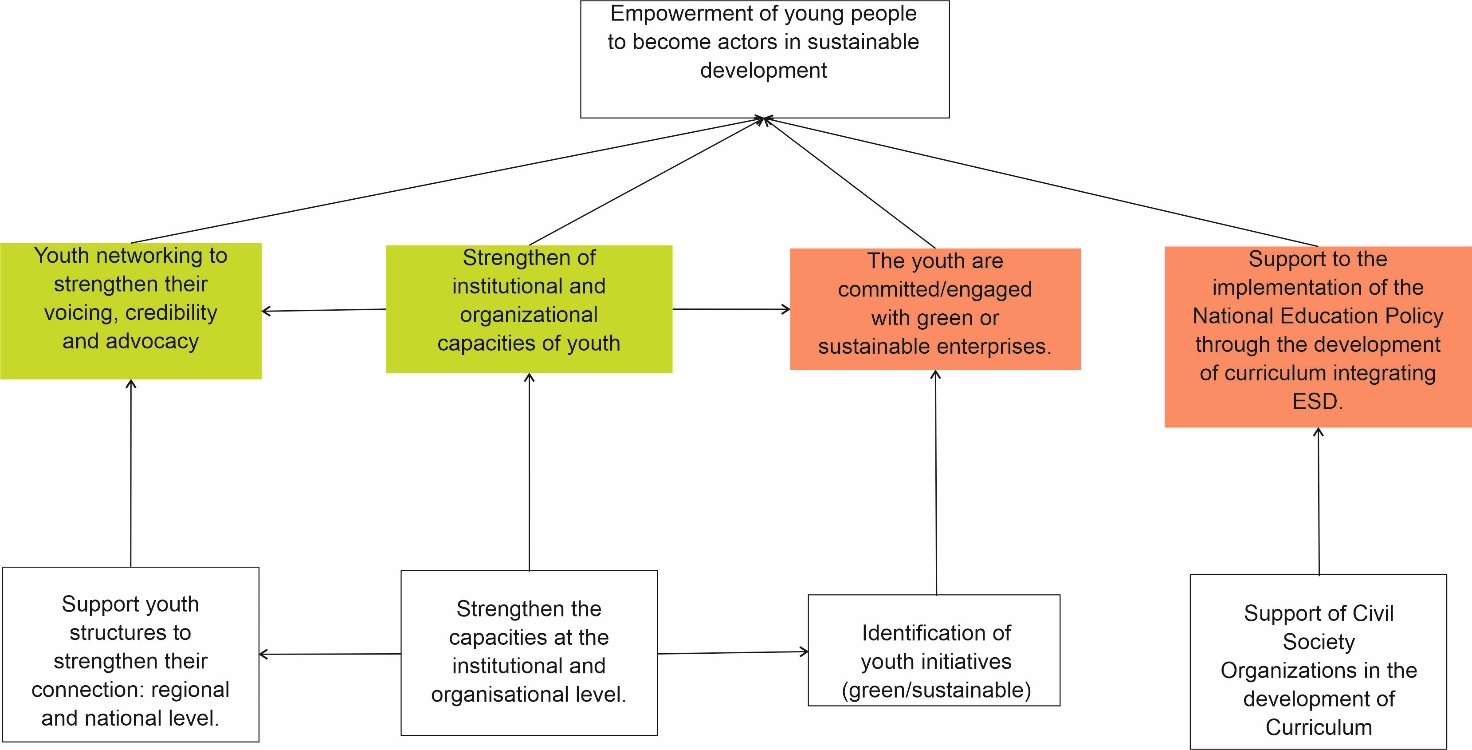


Figure S7F. Individual ToC interviewee F


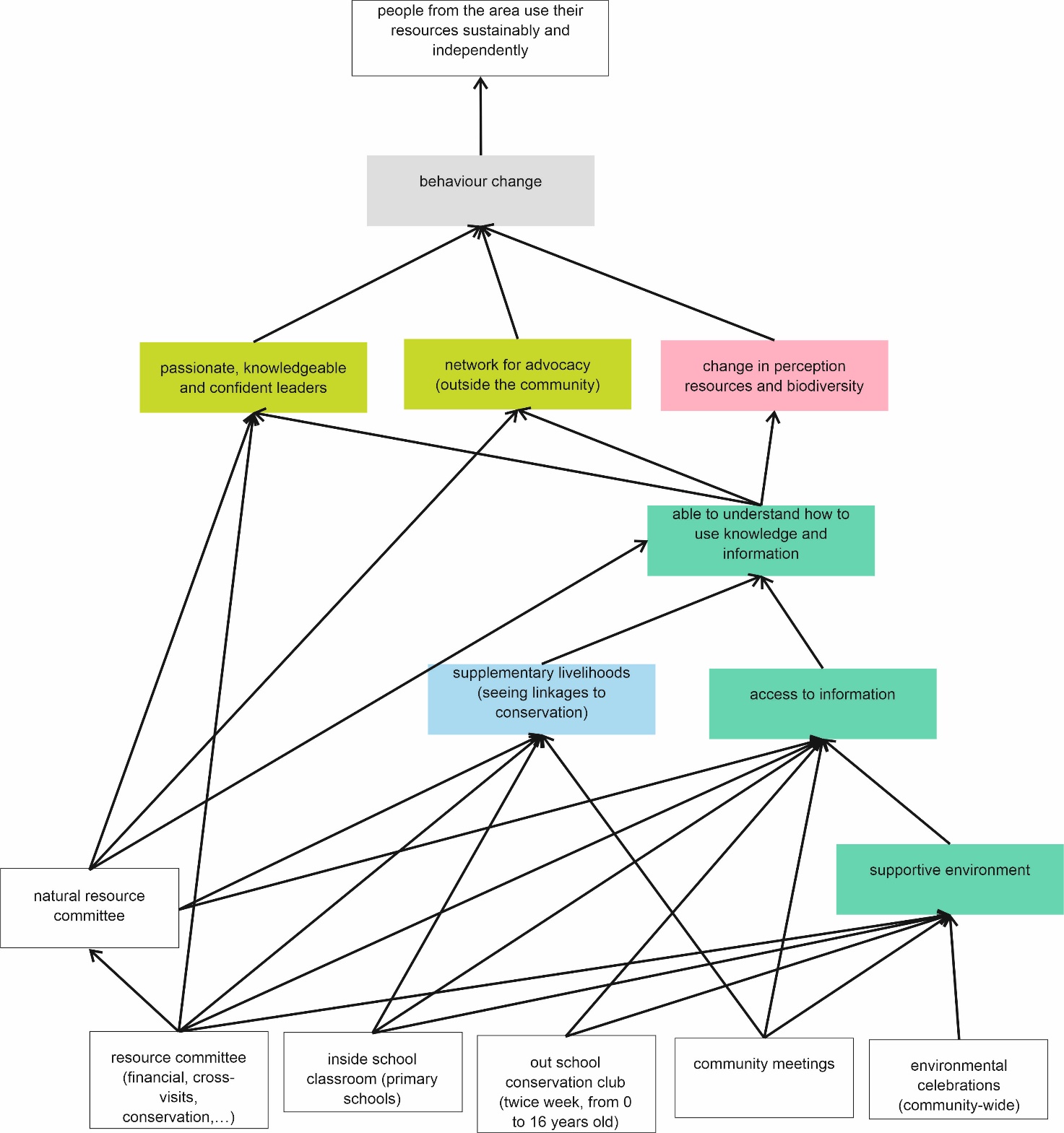


Figure S7G. Individual ToC interviewee G


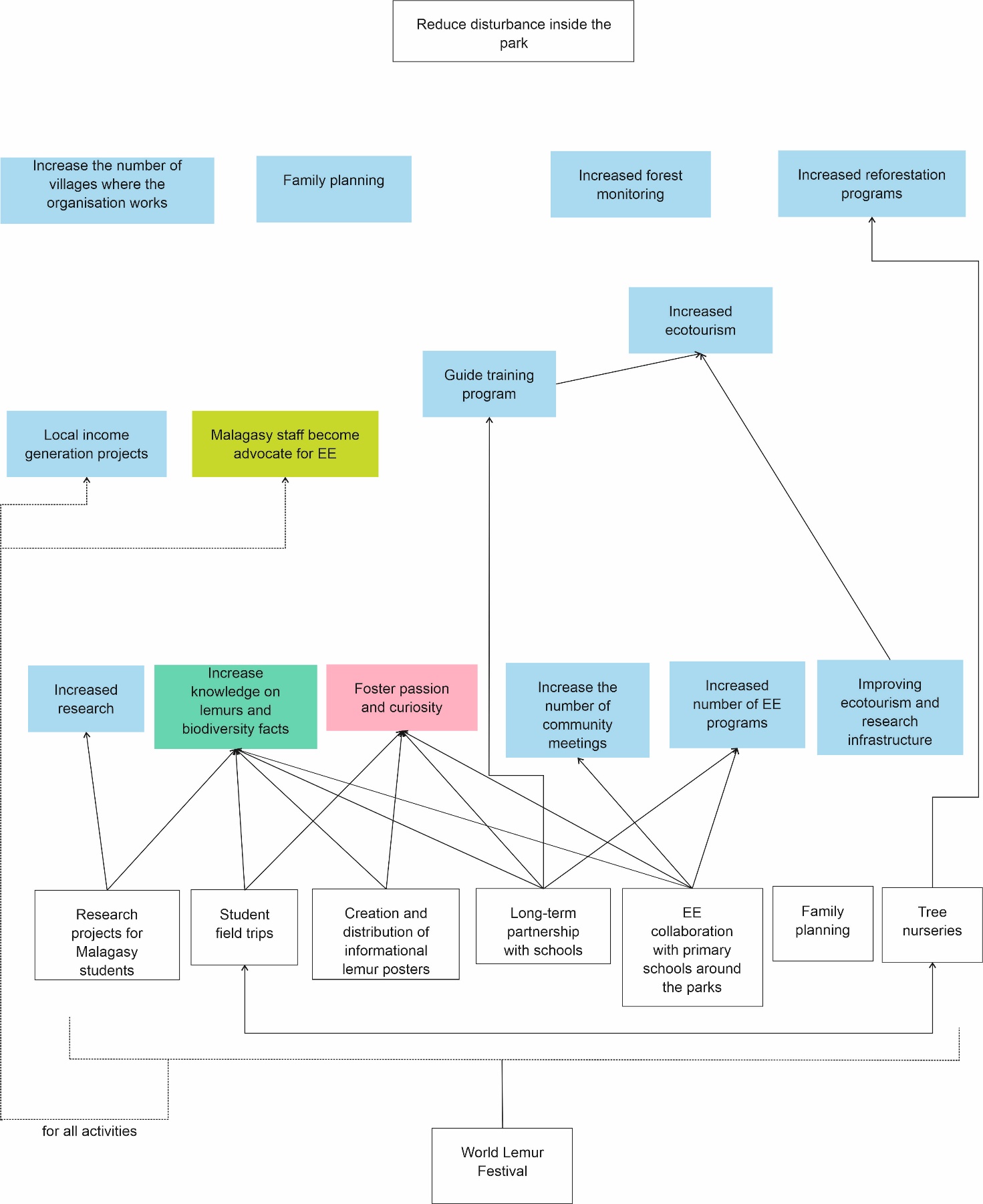


Figure S7H. Individual ToC interviewee H


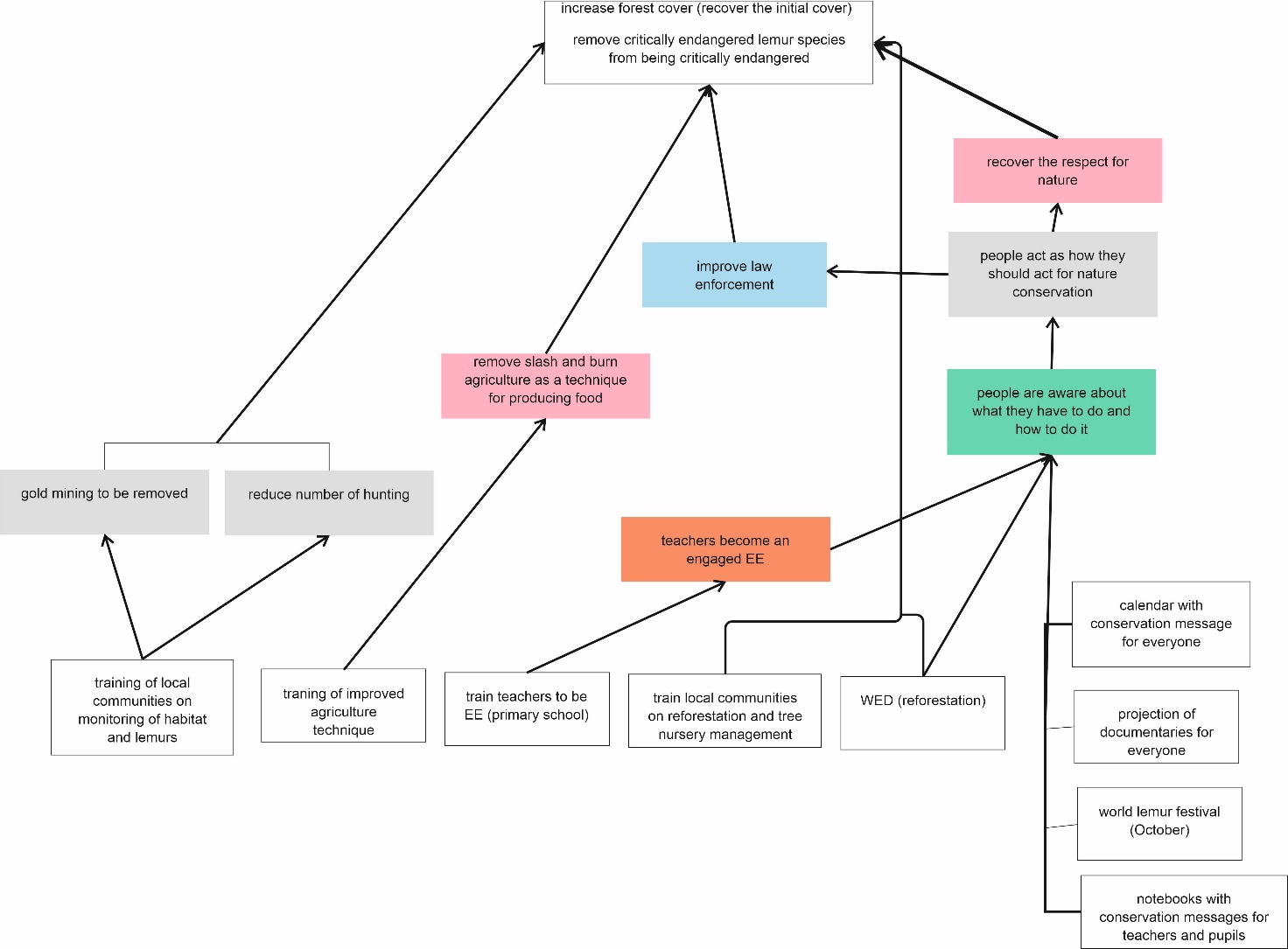


Figure S7I. Individual ToC interviewee I

**
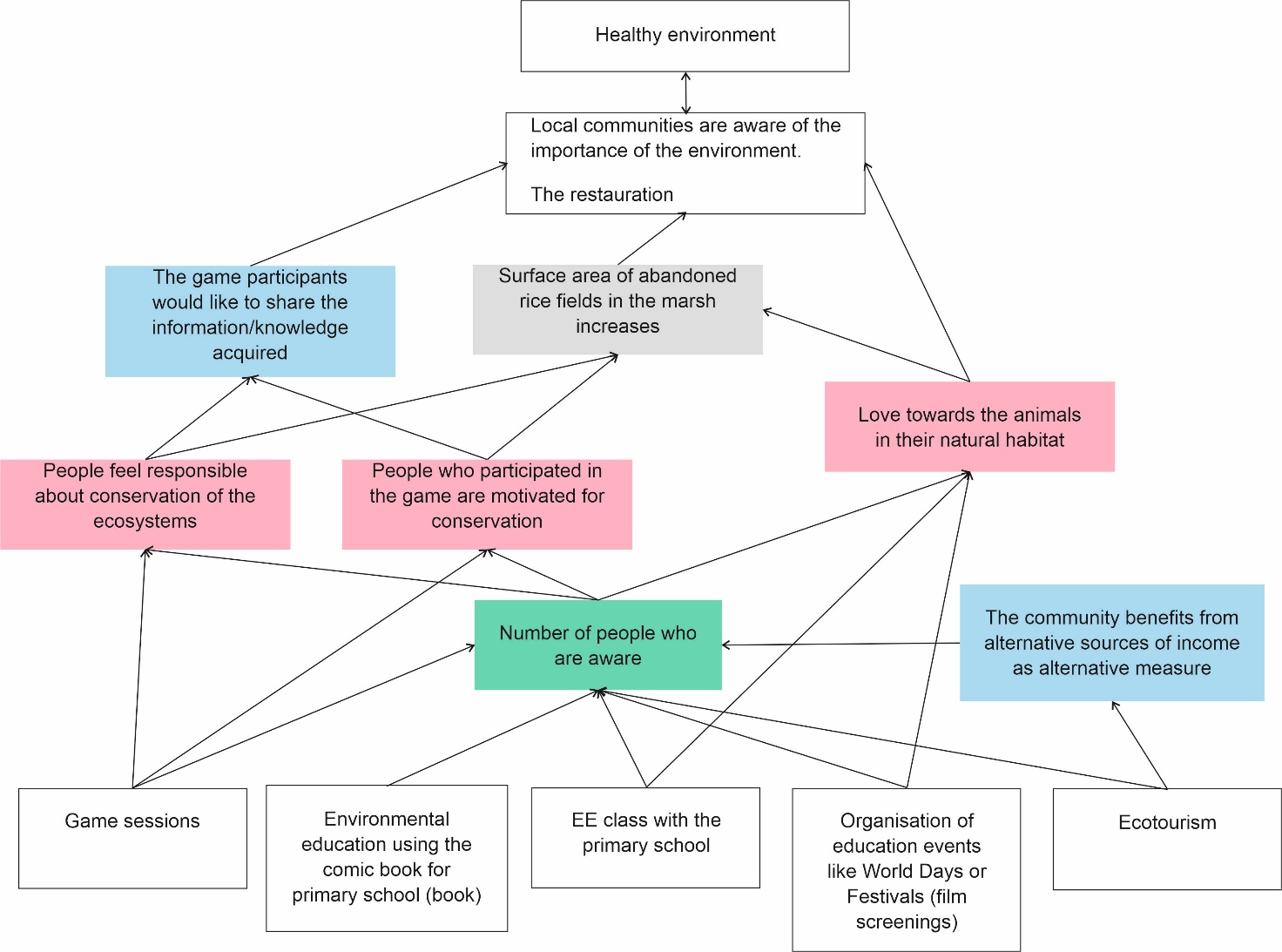
**

Figure S7J. Individual ToC interviewee J

**
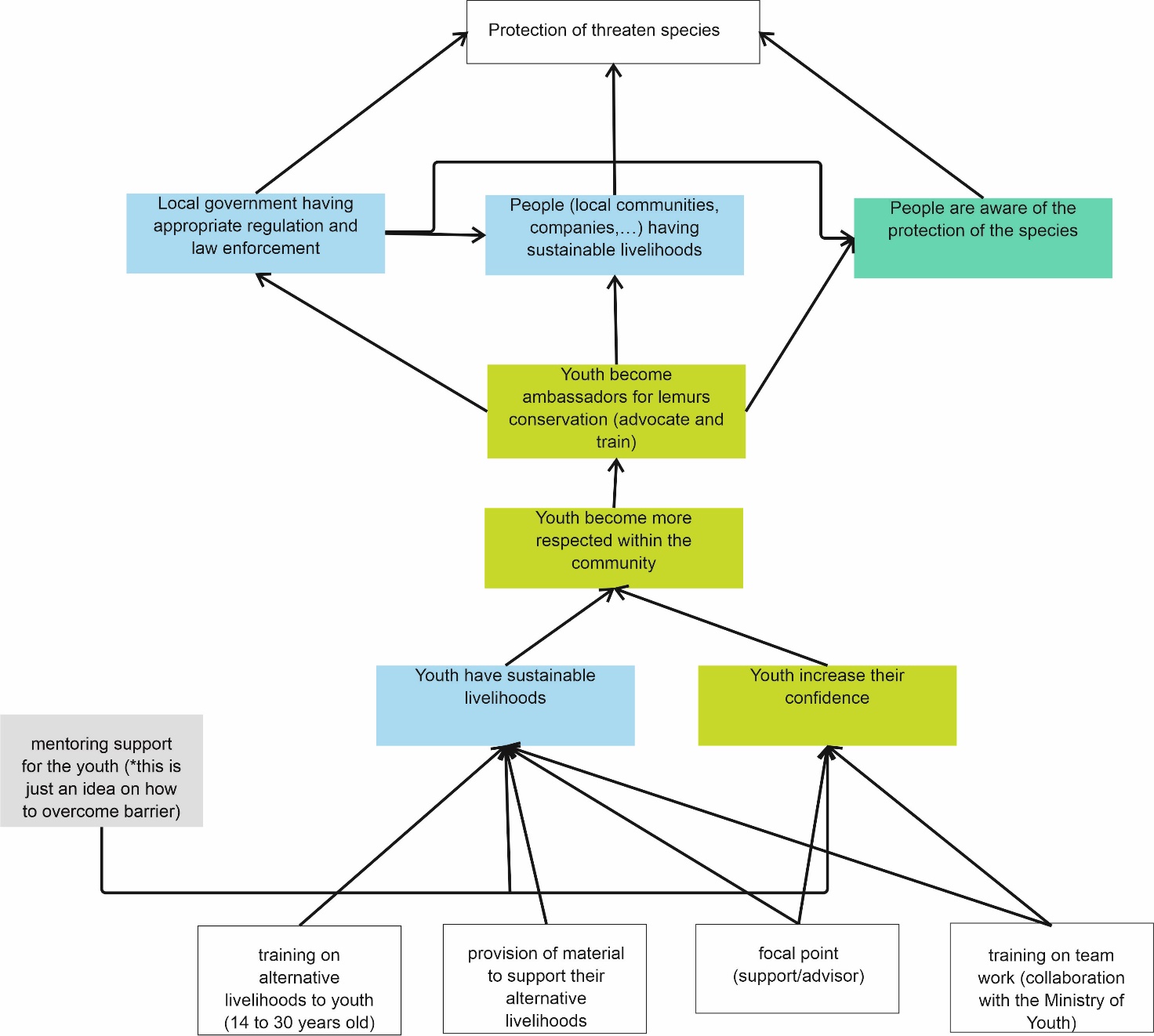
**

Figure S7K. Individual ToC interviewee K

**
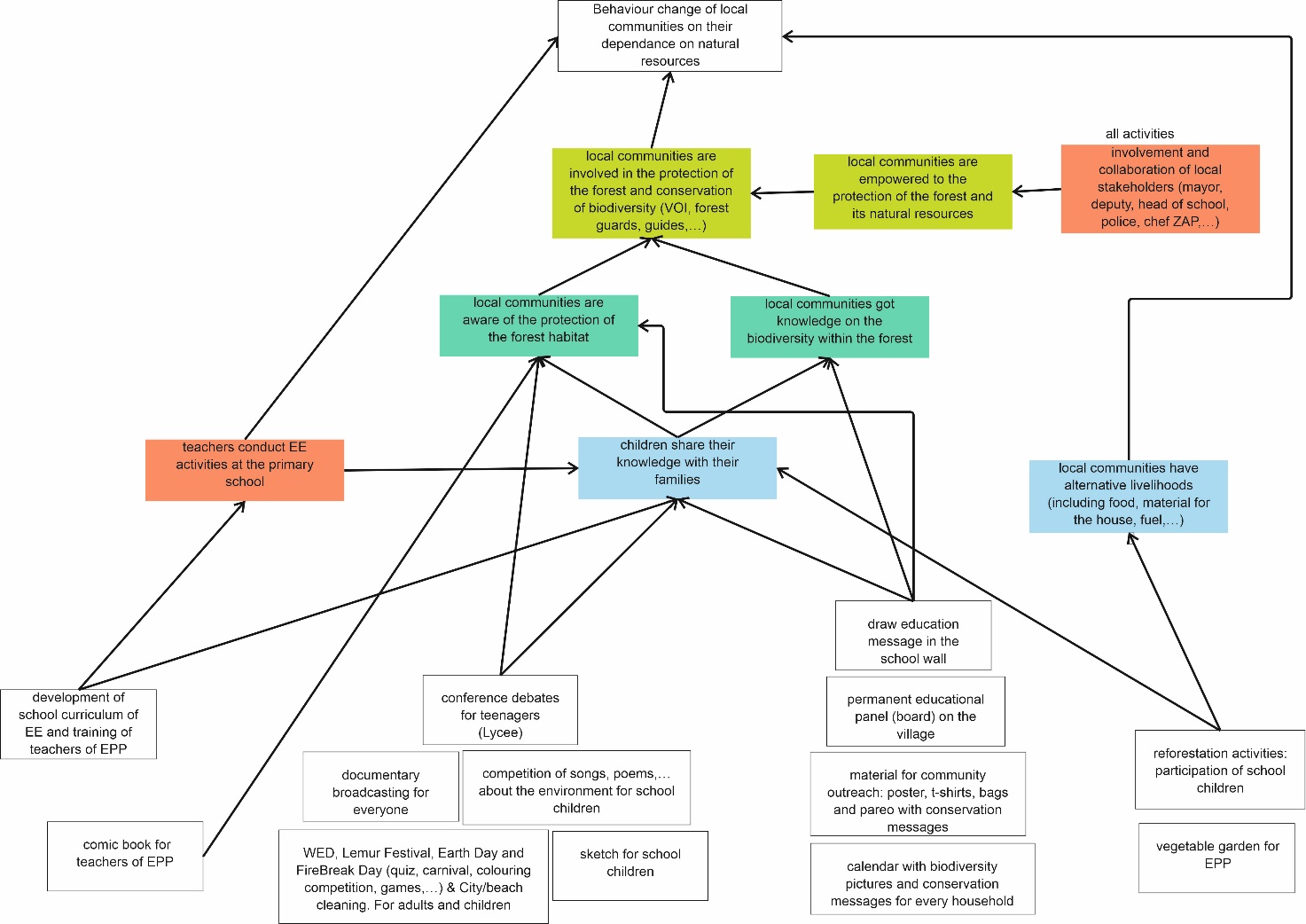
**

Figure S7L. Individual ToC interviewee L

**
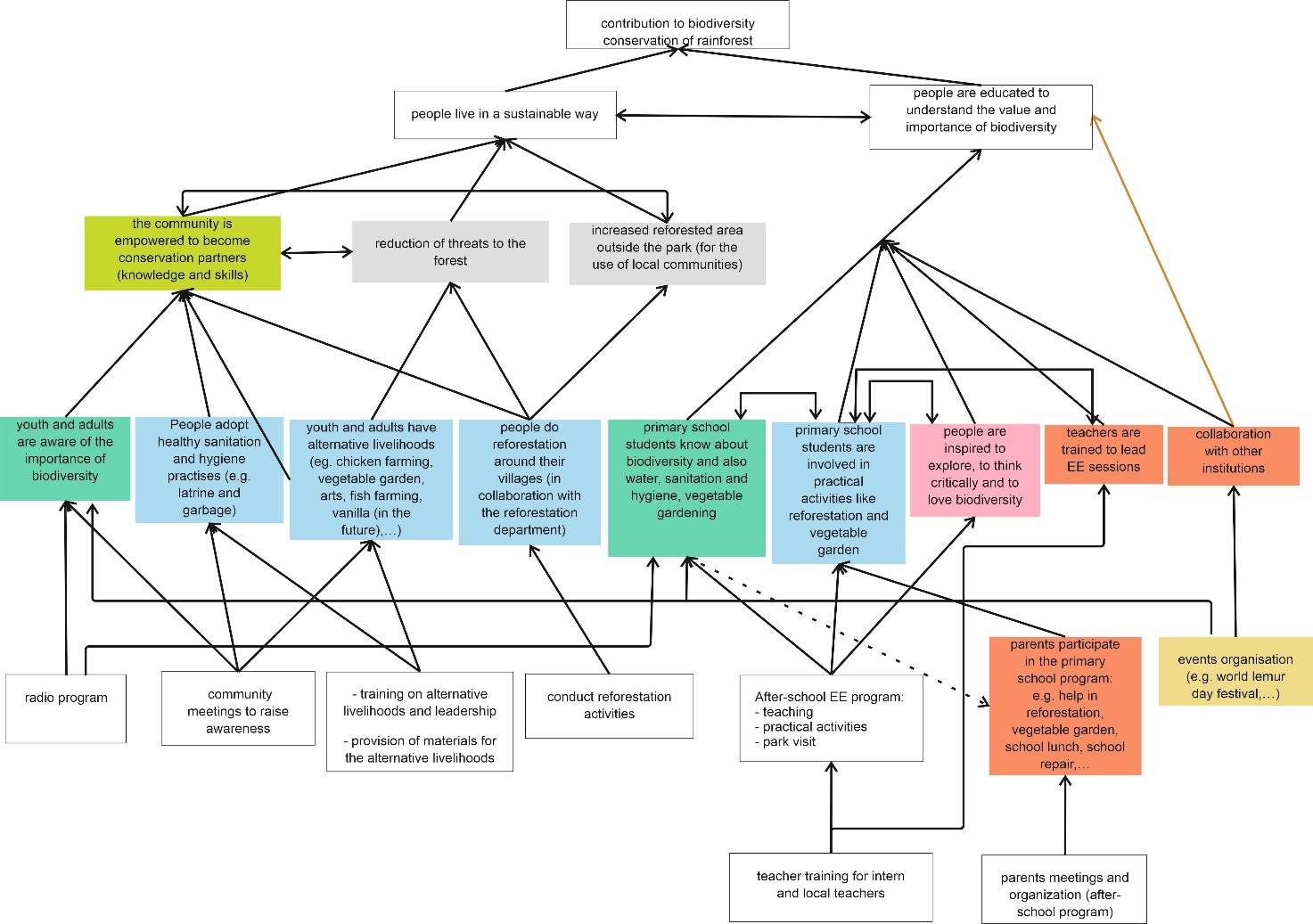
**

Figure S7M. Individual ToC interviewee M

**
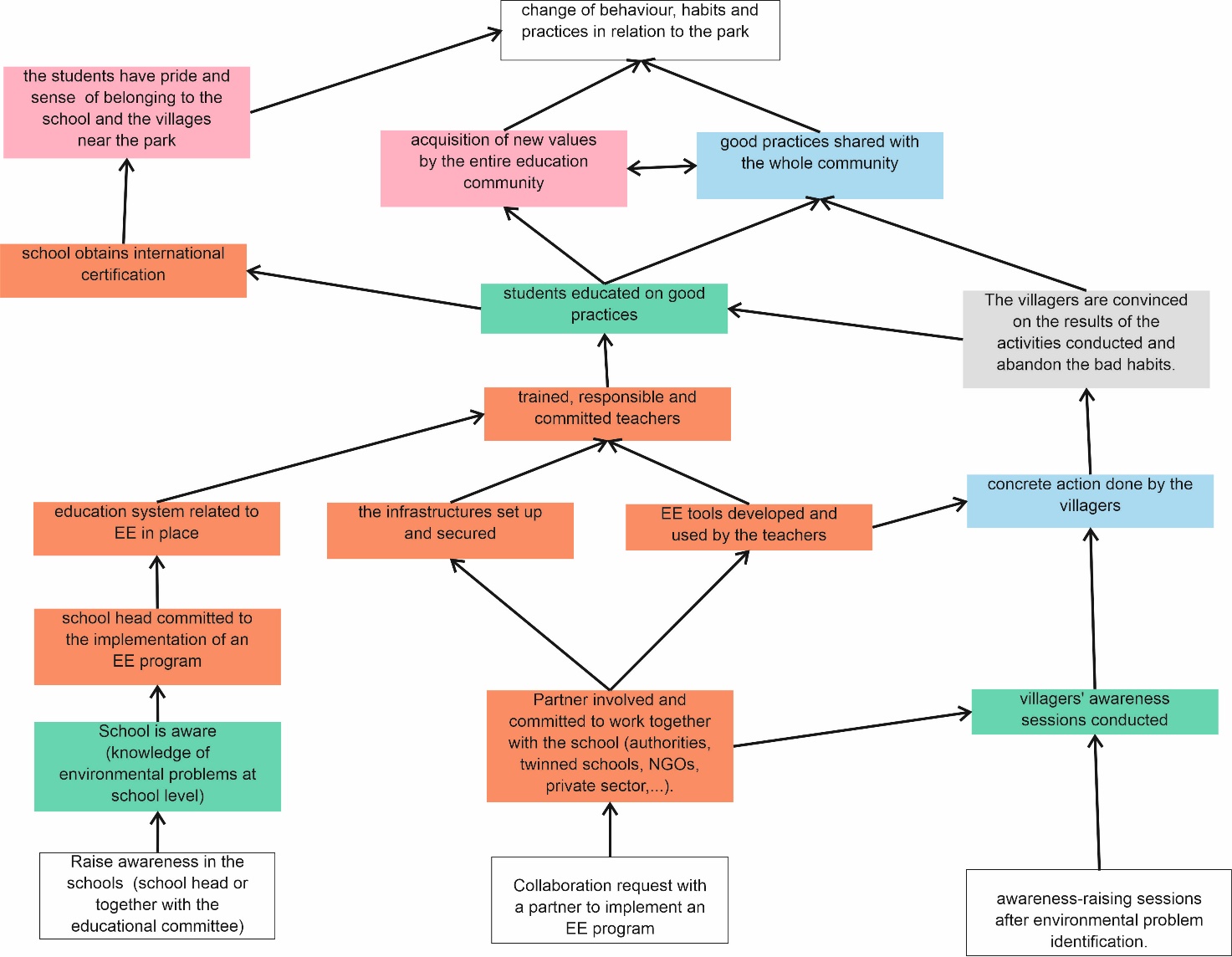
**

Figure S7N. Individual ToC interviewee N


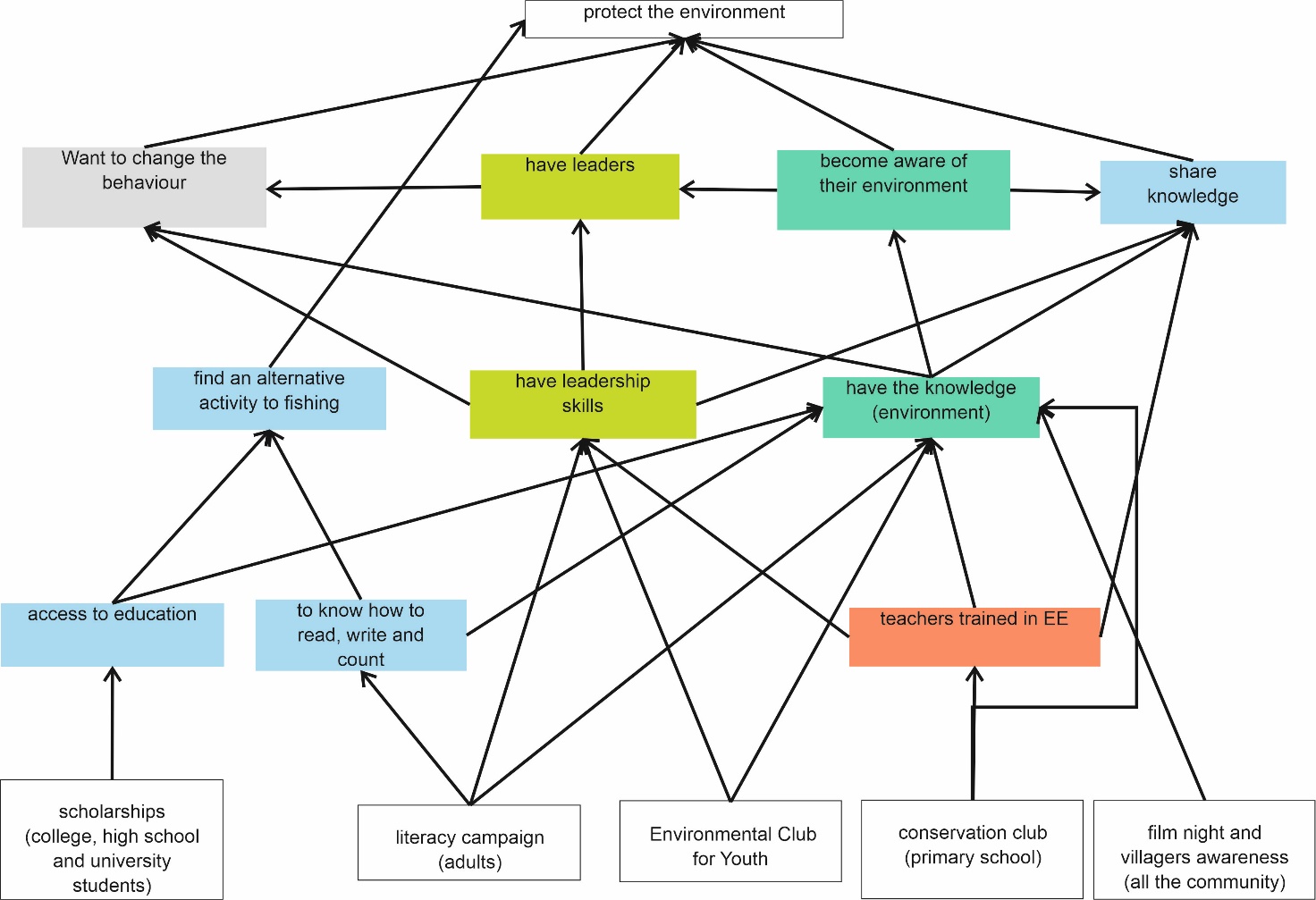


Figure S7O. Individual ToC interviewee O

**Additional references**

Balfour, D., Barichievy, C., Gordon, C., & Brett, R. (2019). A Theory of Change to grow numbers of African rhino at a conservation site. *Conservation Science and Practice*, *1*(6), e40. https://doi.org/10.1111/csp2.40

Belcher, B., & Claus, R. (2020). Theory of Change. Td-net toolbox profile. *Swiss Academies of Arts and Sciences: Td-Net Toolbox for Co-Producing Knowledge*, *5*. https://doi.org/doi.org/10.5281/zenodo.3717451

Center for Theory of Change. (2021). *How Does Theory of Change Work?* Center for Theory of Change. https://www.theoryofchange.org/what-is-theory-of-change/how-does-theory-of-change-work/

LaMere, K., Mäntyniemi, S., Vanhatalo, J., & Haapasaari, P. (2020). Making the most of mental models: Advancing the methodology for mental model elicitation and documentation with expert stakeholders. *Environmental Modelling & Software*, *124*, 104589. https://doi.org/10.1016/j.envsoft.2019.104589

Oppenheimer, D. M., LeBoeuf, R. A., & Brewer, N. T. (2008). Anchors aweigh: A demonstration of cross-modality anchoring and magnitude priming. *Cognition*, *106*(1), 13–26. https://doi.org/10.1016/j.cognition.2006.12.008
